# Activity of Selected Group of Monoterpenes in Alzheimer’s Disease Symptoms in Experimental Model Studies—A Non-Systematic Review

**DOI:** 10.3390/ijms22147366

**Published:** 2021-07-08

**Authors:** Karolina Wojtunik-Kulesza, Monika Rudkowska, Kamila Kasprzak-Drozd, Anna Oniszczuk, Kinga Borowicz-Reutt

**Affiliations:** 1 Department of Inorganic Chemistry, Medical University of Lublin, Chodźki 4a, 20-093 Lublin, Poland; anna.oniszczuk@umlub.pl; 2 Independent Experimental Neuropathophysiology Unit, Medical University of Lublin, Jaczewskiego 8b, 20-090 Lublin, Poland; monika.rudkowska@umlub.pl (M.R.); kinga.borowicz-reutt@umlub.pl (K.B.-R.)

**Keywords:** monoterpenes, Alzheimer’s disease, memory, anxiolytic activity, sleep regulatory, insomnia

## Abstract

Alzheimer’s disease (AD) is the leading cause of dementia and cognitive function impairment. The multi-faced character of AD requires new drug solutions based on substances that incorporate a wide range of activities. Antioxidants, AChE/BChE inhibitors, BACE1, or anti-amyloid platelet aggregation substances are most desirable because they improve cognition with minimal side effects. Plant secondary metabolites, used in traditional medicine and pharmacy, are promising. Among these are the monoterpenes—low-molecular compounds with anti-inflammatory, antioxidant, enzyme inhibitory, analgesic, sedative, as well as other biological properties. The presented review focuses on the pathophysiology of AD and a selected group of anti-neurodegenerative monoterpenes and monoterpenoids for which possible mechanisms of action have been explained. The main body of the article focuses on monoterpenes that have shown improved memory and learning, anxiolytic and sleep-regulating effects as determined by in vitro and in silico tests—followed by validation in in vivo models.

## 1. Introduction

“Aging and death do seem to be what Nature has planned for us. But what if we have other plans?”—Bernard Strehler.

Dementia and neurodegeneration are unfortunately common in the world of today. Indeed, in accordance with the World-Alzheimer report 2019, over 50 million patients globally suffer from Alzheimer’s disease (AD), the most important cause of dementia. Long-lasting studies have revealed the multi-factorial nature of a majority of such disorders, and current research has attracted a wide circle of scientists specializing in various branches of life science.

The characteristic features of AD can be divided into early, middle-stage and later symptoms. Overall, however, AD is an insidious disorder, the first symptoms of which are non-specific. The first, e.g., misplacing items or forgetting recent conversations, are particularly hard to identify [1]. Acknowledgement of this fact can be considered a key moment in diagnosis that had influence on treatment success, as the mysterious attributes of the disease brought about attention to the molecular basis of it, with the result being new treatment regimes and improvement in the currently available drugs.

It is commonly recognized that AD and numerous other neurodegenerative diseases exhibit a multi-factorial nature. Therefore, the most promising treatment pathways are based on multi-directed substances. To date, FDA-approved pharmacological treatment includes inhibitors of acetylcholinesterase and an NMDA receptor antagonist [2]. Nevertheless, weak treatment effectiveness, along with adverse side effects, has compelled a search for new drug solutions.

One of the latest directions of study lies in traditional plant-based medicine. Indeed, many plant-derived active substances are in current use as pharmaceuticals. Among these are the terpenes. These secondary plant metabolites demonstrate various biological activities, including antioxidant, enzyme inhibitory (acetylcholine esterase—AChE, amylase, glucosidase), antifungal, hepatoprotective, or sedative [1,3,4,5,6].

The monoterpenes are a terpene subgroup and are isoprene derivatives responsible for the aromatic character of plants. These secondary metabolites are synthesized in response to biotic and abiotic stress. Low molecular isoprenoids are mostly ubiquitous in plants that have evolved to survive in stressful conditions (e.g., exposure to high doses of UV radiation) [7]. Such substances have been identified in various plants families (e.g., *Lamiaceae*), including the citrus species. Due to the fact that terpenes are characterized by their aromatic flavor, plants rich in the substances are used as food additives and aromatherapy products.

From the chemical point of view, monoterpenes can be divided into three groups: 1. acyclic monoterpenes (e.g., citral, citronellal), 2. monocyclic monoterpenes (e.g., menthol, carvone), and 3. bi- and tricyclic terpenes (e.g., nepetalactone, santonin). Groups 1 and 2 are the most intensively studied due to the fact that they are exemplified by their low molecular character. The numerous biological activities of such compounds have been revealed in in vitro studies (e.g., antioxidant, antiviral, sedative, skin permeation enhancement) and confirmed in in silico and/or in vivo assays [8,9].

Among the most intensively studied drug-based activities against neurodegeneration, including AD, lie in memory and learning enhancement, anxiolytic and insomnia countermeasures. Successful research of the problem is usually verified with use of in vivo models after initial in vitro and in silico experiments.

The presented review will focus on the effects of selected monoterpenes and essential oils activities against AD factors and symptoms in in vitro, in silico, and mouse/rat models.

## 2. Pathophysiology of Alzheimer’s Disease

AD is a neurodegenerative disorder characterized by two important neuropathological features: extracellular senile plaques—which are mainly composed of β-amyloid (Aβ) deposits, and intracellular neurofibrillary tangles (NFT)—consisting of bundles of paired helical filaments (PHF), the main component of which is a pathologically hyperphosphorylated tau protein (hptau) [10]. Senile plaques and tangles accumulate mainly in the hippocampus, amygdala, entorhinal cortex and basal part of the forebrain, i.e., the brain areas responsible for memory, learning, and emotional behaviors [11]. Loss of synapses and neurons are other commonly accepted pathological features of AD [10].

### 2.1. β-Amyloid

Aβ peptides accrue as a result of the abnormal proteolytic processing of the amyloid precursor protein (APP), which is found in most tissues in the body [12]. Physiologically, APP regulates synaptic formation and repair, iron export and anterograde neuronal transport [13,14]. APP is a transmembrane protein and consists of three fragments: the N-terminal long extracellular segment, the short endothelial region, and the short C-terminal fragment found in the cytoplasm [15]. Two enzymes affect the APP protein: α-secretase (TACE) and β-secretase (BACE1). These enzymes split the extracellular fragment of APP, which results in the production of two soluble N-terminal peptides (sAPPα and sAPPβ, respectively) and two C-terminal segments CTFα (C83) and CTFβ (C99) that are bound to the cell membrane. Subsequently, CTFα and CTFβ are cleaved by γ-secretase inside the membrane. As a result, the soluble p3 peptide, which has no tendency to aggregate, is released from CTFα. In turn, the breakdown of CTFβ leads to formation of Aβ (released into the extracellular space) and the intracellular domain (AICD). AICD can travel to the nucleus, where it can affect gene expression and induce cellular apoptosis [16,17]. Aβ can be constructed from 40–42 amino acids, depending on where the γ-secretase cleaves the protein chain [18,19]. The Aβ protein exists in two different forms: non-pathogenic Aβ40 protein and protein Aβ42. The last is hydrophobic, can easily aggregate, and is the main component of amyloid plaques [20]. Aβ42 is mainly produced by microglial cells and astrocytes [21].

Increased neuronal activity of the brain and stimulation of muscarinic acetylcholine receptors enhance the activity of α-secretase. These effects correlate with an increase in sAPPα levels [22]. The N-terminal peptide sAPPα positively influences the plasticity of the brain by stimulating the growth of neuronal progenitor cells. Therefore, neurodegeneration in AD may also result from decreased sAPPα levels [23]. In contrast, sAPPβ contributes to neuronal death and axon pruning, and its increased concentration in the gray matter surrounding neuritic plaques and cerebral blood vessels has been observed in patients with AD [24,25].

Presenilins (PSs) exist in the form of two highly homologous proteins, PS1 and PS2, and are involved in APP proteolysis. PSs are transmembrane proteins that are found mainly in nerve cells, where they can act as membrane receptors or calcium channels. PSs increase the susceptibility to apoptosis and regulate calcium homeostasis in the endoplasmic reticulum (ER) [26,27]. Hippocampal and Purkinje cells exhibit the strongest expression of PSs. It has been suggested that PS1 may act as a γ-secretase or γ-secretase cofactor [28,29]. Mutations in the presenilin genes are a significant cause of the formation of the Aβ40 and Aβ42 [27].

Under normal conditions, Aβ can modulate neurotransmission and substantially increase potentiation in the hippocampus [30,31]. Aβ exists as Aβ monomers of various forms that aggregate to form oligomers (soluble forms of Aβ), and then protofibrils and amyloid fibrils (an insoluble form of Aβ which forms amyloid plaques) [32]. It is possible, however, that the fibers arise directly from the monomers—without the structure of the oligomers [33]. Each of these amyloid forms can induce neurotoxic effects at different stages of AD [34].

Aβ aggregation leads to changes in the kinase/phosphatase activity and consequently to the hyperphosphorylation of the tau protein and the formation of NFTs [32]. Moreover, the formation of aggregates leads to the generation of free radicals as reactive oxygen species (ROS), which are the cause of the formation of oxidized proteins and peroxided lipids. These, in turn, bring about a number of unfavorable changes in cellular activity, including: ion-motive ATPases dysfunction, impaired function of glucose and glutamate transporters, Ca^2+^ homeostasis disorders, dysfunction of various signaling pathways and even DNA oxidation [35,36,37]. Additionally, increased concentration and aggregation of Aβ stimulates the response of the innate immune system: it activates phagocytosis and immune receptors. In addition, it initiates the development of an inflammatory reaction and the release of inflammatory mediators [38,39,40]. What is more, the APP protein located on the mitochondrial membrane induces mitochondrial dysfunction, which in turn generates an increase in Aβ production [41]. All of these processes can lead to neuronal death and reduction of the number of synapses in the brain. Aβ mainly destroys the nerve cells that synthesize serotonin and norepinephrine and that use glutamate or acetylcholine as neurotransmitters [42].

Soluble forms of amyloid can interact with cell surface receptors and bind to a variety of extracellular molecules, metals, and cell membranes, contributing to their toxicity. Aβ oligomers can bind to receptors such as the Aβ-binding p75 neurotrophin receptor (P75NRT), the low-density lipoprotein receptor-related protein (LRP), cellular prion protein (PrPc), metabotropic glutamate receptors (mGluR5), *α* subunit containing nicotinic acetylcholine receptor (α7nAChR), N-methyl-D-aspartic acid receptor (NMDAR), β-adrenergic receptor (β-AR), erythropoietin-producing hepatoma cell line receptor (EphR), and paired immunoglobulin-like receptor B (PirB). This results in the activation of downstream pathways, leading to impaired mitochondrial function, oxidative stress, and, consequently, Ca^2+^ influx and neurotoxicity [43]. The generation of the hptau or the induction of an inflammatory reaction may also be the result of interaction of Aβ with potential receptors [43,44].

Brain Aβ levels are dependent on a number of processes, including production, degradation, cell-mediated clearance, transport across the blood brain barrier (BBB), and accumulation in insoluble aggregates. One of the most important is the Aβ degradation by various proteases. Neprilysin (NEP) plays a major role in the decomposition of Aβ. It is a zinc metallo-endopeptdase involved in the breakdown of many bioactive peptides [45]. Hence, decreasing NEP activity that progresses with aging may be the cause of idiopathic AD. Endothelin converting enzymes 1 and 2 (ECE1 and ECE2) have also been shown to reduce amyloid levels [46]. Furthermore, insulin-degrading enzyme (IDE) has been revealed to have an important role in degrading many substrates, including insulin, glucagon, amylin, and other bioactive peptides. IDE is a protease that acts directly on Aβ, but also on the intracellular domain of APP (AICD), thus indirectly regulating the level of amyloid. Plasmin can breakdown and reduce the toxicity of both monomer and fibril forms of Aβ [47,48]. Other potential proteases that degrade Aβ in vitro are: matrix metalloproteases MMP2 and MMP9 [49], angiotensin-converting enzyme [50], and cathepsin D [51].

Aβ is synthesized in the peripheral tissues and in the central nervous system. Present in the extracellular space, it is exchanged between the brain and blood. This process is regulated by various receptors. The advanced glycation end products (RAGE) receptor is responsible for the transport of Aβ from the blood to the brain, while low-density lipoprotein receptor-related protein 1 (LRP-1) determines the flow of amyloid from the cerebrospinal fluid to the blood [52,53,54]. Glycoprotein 330 (gp330/megalin) and P-glycoprotein are also involved in the transport of Aβ across the BBB [55]. In addition, transport proteins that bind to Aβ, such as α2-macroglobulin, apoE, and apoJ, may play an important role in this process [56].

Over the years, the functional capacity of the BBB weakens, while inflammatory processes may disturb its continuity. Furthermore, damage to BBB structure may accelerate amyloid deposition by influx of Aβ40 and Aβ42 molecules from peripheral blood to the brain [57,58]. The pathogenesis of AD is still not, however, fully understood. Amyloid hypothesis does not explain all cases of the disease and there is evidence that plaque quantity in control groups is comparable to their number in patients with AD. In addition, it was observed that the number of senile plaques among patients with this neurodegenerative disease poorly correlated with the severity of symptoms [32]. Accordingly, other alternative hypotheses about the causes of this have been proposed.

### 2.2. Tau Protein

The tau protein is expressed in nerve cells. Its physiological function is to stabilize microtubules in the cell cytoskeleton. Moreover, tau allows the transport of signaling molecules, trophic factors, etc. along the axon [59]. Under normal physiological conditions, tau is in constant dynamic balance. This protein binds briefly to microtubules and then is phosphorylated by kinases, which bring about a short-term detachment from microtubules. After dephosphorylation by phosphatases, it is reattached to microtubules. Frequent cycles of such attachment and detachment are essential for effective axonal transport [60].

The hyperphosphorylated form of tau shows less affinity for microtubules and it aggregates more easily to form PHFs and NFTs inside neurons [61]. The result is a disturbance of the function of microtubules, axonal transport, degradation of the cytoskeleton of the cell, and, consequently, damage to the nerve cells. Formation of pathological forms of tau protein can be triggered by Aβ. On the other hand, hptau can stimulate Aβ synthesis through a feedback loop mechanism [62]. Toxic tau can also arise under the influence of oxidative stress, an inflammatory reaction in the nervous system and enzymes modulating kinase and phosphatase activity [63]. Perhaps the most important enzymes are: glycogen synthase kinase 3 (GSK 3), cyclin-dependent kinase 5 (CDK5), and microtubule affinity-regulating kinase (MARK) [64]. There are two forms of GSK: GSK3α and GSK3β. GSK3β is involved in tau phosphorylation, while GSK3α can regulate APP protein processing and Aβ formation. GSK3 promotes the production of inflammatory molecules and the microglia response to inflammation. Therefore, it appears that GSK3 may be involved not only in the primary pathological processes underlying AD, but also in inflammatory processes in the nervous system [65]. CDK5 also participates in tau phosphorylation and NFTs formation, and may regulate GSK3 activity [66].

### 2.3. Oxidative Stress

The equilibrium state between the rate of ROS production and the concentration of low molecular weight antioxidants and enzymes responsible for their decomposition determine the concentration of ROS and the rate of reactions induced by them. Releasing too much ROS causes oxidative stress—a state in which the increasing level of free radicals and their reaction products outweighs the possibility of their decomposition [67]. Oxidative stress plays an important role in the pathogenesis of neurodegenerative diseases.

Free oxygen radicals initiate an inflammatory reaction, but also trigger transcription of genes of various inflammatory factors [68]. The adverse effect of ROS leads to inactivation of proteins containing thiol groups, inhibition of glycolysis by inactivation of glyceraldehyde 3-phosphate dehydrogenase, and to DNA damage. ROS oxidizes proteins. This subsequently leads to their chemical modifications. Moreover, ROS contribute to lipid peroxidation, which causes disturbances in the ion potential of the cell membrane and, as a result, leads to its damage [69].

Cells use endogenous systems to protect themselves from the adverse effects of ROS. These include enzymes such as: glutathione peroxidase, glutathione reductase and superoxide dismutases—located in the cytoplasm (FeSOD and Cu/Zn SOD), and catalase, myeloperoxidase, and a very important complex of NADPH oxidase—located in the mitochondrial matrix (MnSOD) [70]. The brain is particularly susceptible to oxidative damage because it is rich in lipids, including unsaturated fatty acids. Additionally, certain regions of the human brain contain significant amounts of metal ions, especially Fe^3+^, Cu^2+^, and Zn^2+^. These promote the formation of ROS [71]. The effects of reactive oxygen species are considered one of the main causes of neurodegenerative diseases. Studies have shown that senile plaques influence the secretion of excessive amounts of hydrogen peroxide, induce peroxidation of cell membrane lipids and activate the NADPH-dependent microglia oxidase complex and SOD. As a result of lipid peroxidation, 4-hydroxynonenal (among others) is formed. The high toxicity of this is due to it inhibiting the activity of antioxidant enzymes such as glutathione peroxidase and catalase. Additionally, 4-hydroxynonenal is associated with the formation of NFTs. Increased SOD activity and decreased glutathione transferase activity, which participates in the neutralization of 4-hydroxynonenal, are also observed [67,69,72,73].

### 2.4. Inflammation

Encephalitis is an important factor in the pathogenesis of AD. The cells involved in the inflammatory response in the CNS are microglia, astrocytes, and neurons. These, when activated by various factors, can produce significant amounts of inflammatory mediators, including cytokines and chemokines [72].

Short-term inflammatory processes do not affect the activity of neurons and may even have a beneficial effect in the removal of neurotoxins or cellular debris. However, the chronic encephalitis that occurs in AD leads to over-activation of microglia and a marked increase in inflammatory mediators. Hence, continuously activated microglia and astrocytes give way to a further increase in the levels of cytokines and chemokines [73]. Chronic inflammation in AD is a response to Aβ plaque deposition and NFTs formation. Moving forward, inflammatory mediators may influence APP levels and APP processing, leading to increased production of Aβ42. Moreover, inflammation may reduce the formation of sAPPα. Additionally, Aβ can induce the expression of some pro-inflammatory cytokines in glial cells, including interleukin-1β (IL-1β), IL-6, tumor necrosis factor-α (TNF-α) and interferon-γ (IFN-γ), leading to a vicious circle. Here, the released inflammatory factors damage the BBB, which allows the influx of immunologically competent cells from the periphery and contributes to the development of an inflammatory reaction in the CNS in patients with AD [74]. Chronic inflammatory conditions also damage neurons and synapses, leading to the development of neurodegenerative diseases.

### 2.5. Apolipoprotein E

Apolipoprotein E (apoE) is synthesized locally in astrocytes. It exists in three isoforms: apoE2, apoE3, and apoE4. ApoE can regulate the transport, clearance, and aggregation of Aβ. The N-terminal region of apoE in neurons interacts with components of the cytoskeleton of the nerve cell and induces the formation of NFTs [75]. On the other hand, the C-terminal fragments of apoE bind to the Aβ, causing the expansion of amyloid deposits. The apoE2 and apoE3 isoforms participate in the repair of peripheral and CNS neurons, and also play a major role in the regulation of metabolism and distribution of cholesterol in neuronal membranes. The most common isoform is apoE3, while apoE2 reduces the risk of AD. In turn, the presence of the apoE4 allele is associated with the enhancement of amyloidogenesis processes and is responsible for the genetic predisposition to AD, increasing the risk of AD approximately 3 times more than the apoE3 allele. Elevated cholesterol concentration in the membranes of nerve cells enhances Aβ aggregation and induces hyperphosphorylation of the tau protein [76].

### 2.6. The Cholinergic System

**Figure 1:**
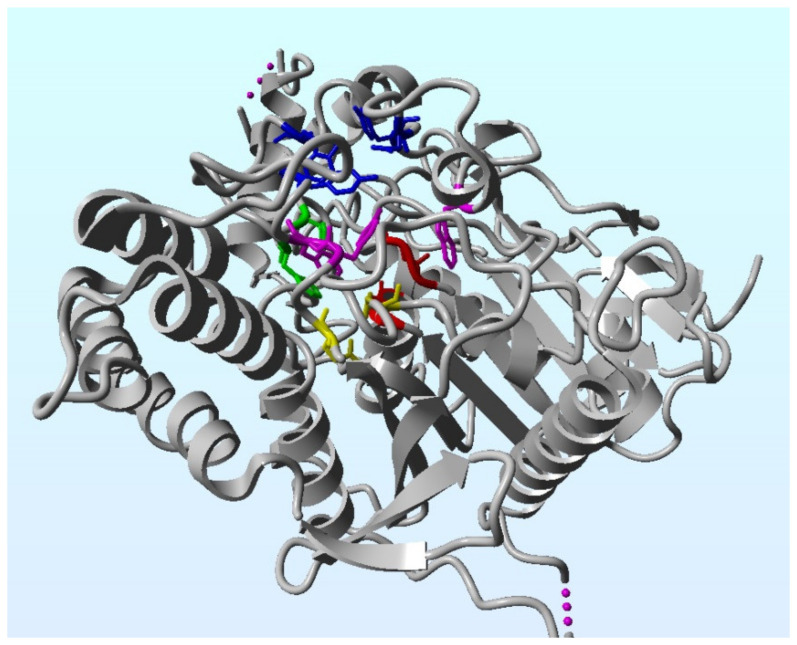
Structure of human acetylcholinesterase (huAChE) with amino acids residues building active site of the enzyme: peripheral anionic site (Tyr341, Trp286, Asp74, Tyr72—blue color), catalytic triad (His447, Glu334, Ser203—yellow color), choline binding site (Trp86, Tyr337, Phe338—magenta color), acyl binding site (Phe 338, Phe295—green color), and anionic site (Gly121, Gly120, Ala204, Ser203—red color). Figure preparation: PDB ID: 4ey6, Yasara 11.2.15 package (Yasara Bioscience, Graz, Austria).

Acetylcholine (ACh) is the main neurotransmitter in the brain. Produced by cholinergic neurons, which are numerous in the CNS (central nervous system), it regulates many cognitive functions, including learning, memory, attention, and thinking abilities [77]. ACh is synthesized by choline acetyltransferase (ChAT) from choline and acetyl-coenzyme A. It is stored in synaptic vesicles and is released into the synaptic cleft as a result of depolarization of the presynaptic membrane. There, it is a ligand for muscarinic (mAChR) or nicotinic (nAChR) receptors located in the postsynaptic membrane, and its excess is broken down into choline and acetate by AChE (Figure 1) [78].

Disturbances in cholinergic transmission associated with altered levels of cholinergic markers such as ACh, choline and ChAT have been found in patients with age-related cognitive impairment in the course of AD. These observations formed the basis of a cholinergic hypothesis that tries to explain the pathogenesis of AD. Disturbances in cholinergic transmission are characteristic of the late stages of neurodegeneration. For example, decreased ChAT and AChE activity was observed in AD, and the use of AChE inhibitors improved cognition in these patients, confirming the role of diminished ACh levels in the pathogenesis of AD [79,80,81].

Additionally, AD patients have a reduced number of nAChR and mAChR receptors. The most important receptor types in AD among nAChRs seem to be α7 nAChR and α4β2 nAChRs, which have been found to be lessened in AD patients [79] (Bekdash, 2021). Stimulation of α7 nAChR in microglia leads to the activation of an anti-inflammatory response and to regulation of oxidative stress. These outcomes further emphasize the role of these receptors in the pathogenesis of AD [82]. Moreover, α7 nAChR can bind to Aβ [83], which modulates signaling pathways related to neuroprotection, synaptic plasticity, learning and memory. Studies have shown that reducing the formation of the α7 nAChRs-Aβ complex improves learning and memory, while its increase negatively affects cognitive functions [84]. Human and animal studies have indicated that the interaction of Aβ and AChR is at the basis of Aβ toxicity [85]. In turn, blockade of the mAch receptors in the hippocampus impairs memory. In AD patients, decreased expression of M1-M4 mAChR types has been observed, and, possibly, Aβ plaques impair the ability of mAChR to transmit cholinergic signals [79,86]. In addition, mAChR agonists reduced amyloid plaque formation and tau protein hyperphosphorylation in a mouse model of AD [81,87].

### 2.7. Glutamatergic System

The gutaminergic system also contributes to the development of neurodegenerative processes. Glutamic acid is the most important excitatory neurotransmitter in the CNS, it is a ligand for both ionotropic and metabotropic receptors. Glutamate is at the crossroads of many metabolic pathways and plays an important role in learning and memory functions. Synaptic damage and neuronal death that occur in AD result in decreased activity of glutamatergic neurons. Disruption of this transmission may have negative effects on memory, cognition, and behavior, including cortical processing and the hippocampus. In contrast, AD may overstimulate NMDA receptors, which are specific for glutamate, leading to an increased influx of calcium ions into the cells, and consequently triggering a series of biochemical reactions that activate the caspase system. The consequence of these processes is the death of neurons [88,89].

## 3. Therapeutic Potential of Monoterpenes for Alzheimer’s Disease

Treatment of AD still remains unsatisfactory. Currently available drugs, including AChE inhibitors, NMDA receptor modulators, and neuroprotective agents, can reduce the symptoms or delay the disease progression, but do not interfere with the causes of AD. Monoterpenes and monoterpenoids constitute an intriguing group of drug candidates that can, however, affect the pathogenesis of AD on multiple target levels [90].

### 3.1. Outline of Mechanisms Involved in the Anti-AD Action of Monoterpenes and Monoterpenoids

The therapeutic potential of monoterpenes and monoterpenoids in the treatment of AD is based on: (1) inhibition of Aβ-induced neurotoxicity; (2) decreased tau-protein phosphorylation, thereby NFTs formation; (3) promotion of Aβ clearance; (4) attenuation of oxidative stress by boosting antioxidant defenses; (5) reduction of neuroinflammation; (6) restoration of mitochondrial function; (7) initiation of processes with simultaneous inhibition of pro-apoptotic genes and proteins, as well as AChE inhibition.

The effect of monoterpenes and monoterpenoids on the formation and aggregation of Aβ seems to be below therapeutic relevance. In research, monoterpenes only moderately inhibited β-secretase, with insignificant effect on the α-secretase that is responsible for non-amyloidogenic APP processing. A possible therapeutic approach for AD is upregulation of the Aβ degrading enzymes such as Aβ proteases, LDL receptor-related protein 1, and the apoE systems [91,92]. Among the protease enzymes, the most important seem to be: NEP, the ECE, angiotensin-converting enzyme, and IDE. Monoterpenes and monoterpenoids inhibit aggregation of the hptau through PI3K/Akt-dependent downregulation of GSK-3β. Decreased GSK-3β activity reduces, in turn, mitochondrial ROS generation [93].

Another established mechanism of monoterpene action is likely to be related to their anti-inflammatory effect. Monoterpenes suppress synthesis of the key inflammatory mediators, including TNF-α, IL-1, COX, and NOS. They also regulate pro-inflammatory cytokines, such as NF-κB, thus playing a key role in the AD pathogenesis. Leptin, however, appears to partially regulate monoterpenoid neuroprotection and cognition immune responses [94,95,96].

The usefulness of monoterpenes and their derivatives in the treatment of AD have been intensively investigated via numerous in vitro and in vivo models. Cell lines used for this purpose include SH-SY5Yand PC12 cells, as well a variety of cortical, hippocampal, and microglial cells of human or animal origin. The most frequently employed in vivo models include mice and rats injected intracerebrally with different fragments of Aβ protein, mice treated with D-galactose, the streptozocine-induced model in rats, double and triple transgenic models in mice [97,98,99].

### 3.2. Monoterpenes and Monoterpenoids in the Treatment of AD—Possible Mechanisms of Action

**Figure 2:**
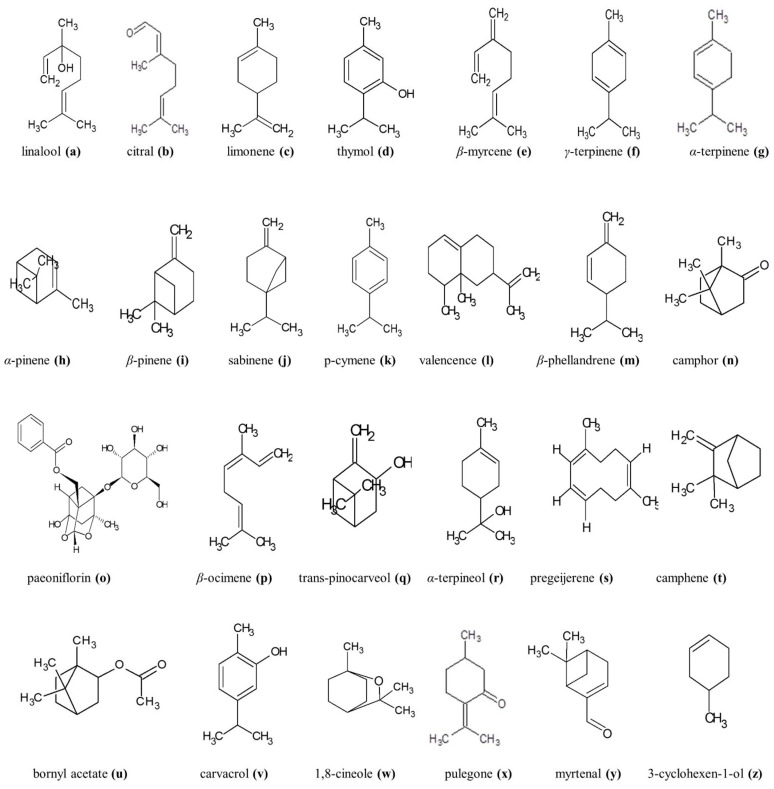
Structures of selected terpenes considered in the AD treatment.

Knowledge about the usefulness of monoterpenes and monoterpenoids in the treatment of AD is rather scarce. Most of the available data concern their influence on AChE activity. Numerous components of essential oils (EOs) extracted from *Salvia lavandulaefolia*, *Pimpinella anisoides*, *Thymus vulgaris*, *Mentha piperita*, *Lavandula luisieri*, or *Nepeta cetaria* inhibit the action of AChE, suggesting their potential for antidementive therapy in AD patients. With regard to this, five compounds have demonstrated the greatest effect. These are myrtenal (**y**), verbenone, carvacrol (**v**), 1,8-cineole (eucalyptol) (**x**), and α-pinene (**h**), with (**z**) being the most active AChE inhibitor. A moderate and rather subtherapeutic effect on AChE was also offered by β-pinene (**i**), α-terpinene (**g**), γ-terpinene (**f**), 3-carene, limonene (**c**), sabinene (**j**), trans-anethole, thymohydroquinone, carvacrol (**v**), thymoquinone, thymol (**d**), linalool (**a**) and pulegone (**x**) [99] (Figure 2). However, such biological substances may serve as template structures to improve activity of existing anti-AD medications. For example, the incorporation of the monoterpene skeleton of camphene (**t**) into the structure of galantamine resulted in the creation of a hybrid that was seen to be over 100 times more active as an AChE inhibitor than galantamine itself [100].

In addition to improving cognitive functions, linalool (**a**) displayed a neuroprotective effect through inhibiting glutamate release and blocking NMDA receptors [101]. In addition, it reversed microgliosis, astrogliosis, tauopathy, β-amyloidosis, and decreased the brain levels of pro-inflammatory markers [102]. In turn, necrodane ketone, geranyl acetone, (**n**) and fenchone moderately inhibited β-secretase, thus decreasing production of Aβ protein. Furthermore, Aβ-induced neurocytotoxicity was reduced by 1,8-cineole, (**h**) and genipin [98,99] (Figure 2).

Studies conducted on mouse models of AD revealed that carvacrol (**v**) significantly reduced the aquaporin-4-dependent brain edema [103]. In contrast, linalool (**a**) normalized the level of some antioxidant enzymes (SOD, GPx) [104] and showed anti-inflammatory action by suppressing levels of pro-inflammatory proteins, e.g., p38 MAPK, NOS-2, COX-2, and IL-1 [102,105].

### 3.3. Iridoids in the Treatment of AD

Iridoids and secoiridoids, both monoterpenoids, possess a broad spectrum of biological activities, including neuroprotection and neuroregeneration. Iridoid glycosides, catalpol (extracted from the *Rehmannia glutinosa* root), and geniposide (a component of *Gardenia jasminoides* fruit) are widely used in traditional Chinese medicine for the treatment of AD [106].

Catalpol seems to be a prospective neuroprotective drug enhancing cognitive functions in AD patients. This iridoid was reported to increase hippocampal cell survival, stimulate neurogenesis, and build-up synaptic plasticity in the mechanism of BDNF levels upregulation. In the hippocampus of aged rats, BDNF, after binding to its specific receptor TrkB, improved expression of synaptophysin, a synaptic vesicle membrane protein, and activated the PKC kinase that in turn increased expression of GAP-43 gene and proteins. It is worth stating that reduced brain levels of BDNF and GAP-43 are considered as biochemical markers of aging and neurodegeneration in AD patients [107,108].

Administration of catalpol also significantly reduced the levels of soluble and deposited Aβ40-42 senile plaques in mouse cerebral neurons. This effect could be due to reduction of ROS levels by increasing brain concentrations of antioxidant enzymes such as SOD, GSHPx, catalase, GSH-ST, glutamine synthetase, and creatine kinase. A second mechanism involves upregulation of IDE expression and, in consequence, increase of Aβ clearance. In the mouse cerebral cortex, catalpol was found to prevent neurodegeneration of choline acetyltransferase-positive cells, enhance the activity of ChAT, increase the number of M1 cholinergic receptors, and decrease the level of inflammatory mediators TNF-α and IL-1 [106].

In vitro studies have revealed that catalpol inhibited Aβ production probably in the mechanism of upregulation of non-amyloidogenic processing of APP. Increased expression of α-secretase through ERK/CREB signaling pathway was noted to reduce APP processing by β-secretase. In a cell model of BBB, catalpol also reduced levels of two matrix metalloproteinases (MMP-2 and MMP-9) and increased those of transporter proteins LRP-1 and P-gp. As a result, this iridoid diminished Aβ-induced membrane hyperpermeability and heightened the clearance of soluble Aβ across the membrane. Furthermore, catalpol regulated the hypothalamic-pituitary-adrenocortical axis and alleviated the structural damage of hypothalamus in an AD rat model [109,110].

In vitro experiments revealed that geniposide increased degradation of soluble Aβ in senile plaques by GLP-1R-dependent upregulation of IDE [110,111]. Interestingly, exendin-4 and liraglutide (antidiabetic drugs being GLP-1R agonists) are under clinical investigation in AD patients [112]. Moreover, geniposide upregulated BDNF and Bcl-2 anti-apoptotic genes in the mechanism of MAPK activation, followed by c-Raf and p90RSK phosphorylation [113].

In the rat hippocampus, geniposide significantly reduced tau hyperphosphorylation through the PI3K/Akt signaling pathway [112]. The molecular mechanism of this phenomenon is supposed to be related to decreased synthesis of the active form of GSK-3β, reported to be responsible for formation of NFTs in AD patients [114].

In another study, geniposide ameliorated mitochondrial dysfunction, restoring the activity of cytochrome c oxidase and ATP generation in mitochondria [115]. What is more, in the mouse cortex and hippocampus, this iridoid ameliorated synaptic injury and improved axonal mitochondrial transport, thus normalizing the levels of several synaptic proteins, e.g., PSD-95 and synaptophysin. In this mechanism, geniposide regulated synaptic plasticity and neurotransmitter release in nerve terminals, hence improving long-term memory storage [116].

In a mouse AD model, geniposide was also found to lower the brain levels of Aβ and to exhibit an anti-inflammatory effect through suppression of RAGE-dependent signaling in activating ERK and IκB/NF-κB. Moreover, it decreased production of TNF-α and IL-1β. RAGE-induced neuroinflammation is suggested to contribute to cognitive dysfunctions in AD mice [117].

In hippocampal neurons of diabetic rats, geniposide was noted to suppress the accumulation of Aβ deposits by reducing expression of β-secretase and to increase clearance of Aβ1-42 deposits by enhanced IDE expression [118]. Both mechanisms are thought to improve synaptic plasticity and cognitive processes [117].

In the mouse model of AD, geniposide triggered the leptin-mediated downregulation of β-secretase, thus decreasing production of Aβ1-42 and its accumulation in senile plaques. Furthermore, leptin suppressed tau phosphorylation via Akt/AMPK-dependent inhibition of GSK-3β [98]. AMPK activation also downregulated β-secretase activity through increased SIRT-1 expression and heightened neuronal glucose uptake via increased expression of GLUT-3 transporter. In brain neurons of AD patients, this mechanism can improve mitochondrial function and reduce Aβ formation through inhibition of the m-TOR pathway [119].

Genipin, an aglycone of geniposide, ameliorated amyloid pathology in in vitro conditions. Moreover, this iridoid inhibited oxidative stress and inflammatory processes in the microglia by decreasing the release of NO, TNF-α, IL-1, PGE-2, and NF-κB. This effect was even greater than that of the paternal geniposide [120,121]. In a rat model of AD, genipin reduced tau phosphorylation and prevented learning deficit [122].

Summing up, the possible mechanisms of the anti-AD action of geniposide and genipin are related to the inhibition of Aβ toxicity, oxidative stress, mitochondrial damage, inflammation, and tau phosphorylation [112].

Harpagoside 5, a major component of *Harpagophytum procumbens* and *Scrophularia ningpoensis*, was noted for decreasing apoptosis of choline acetyltransferase-positive neurons in in vitro conditions. The molecular mechanism of this action is most probably related to the activation of ERK and PI3K/Akt, subsequent upregulation of BDNF levels and downregulation of GSK-3β activity, followed by suppression of the PTEN gene [112]. The last effect is considered as a potential therapeutic target for the treatment of brain injury and cognitive disorders [123].

Morroniside and loganin, iridoid monoterpenoids isolated from *Cornus officinalis* fruit, were discovered to alleviate cognitive impairment, improve synaptic functions, and suppress apoptosis in the hippocampus of AD rats, mostly in the mechanism of increasing levels of neurotrophins. Furthermore, morroniside attenuated tau hyperphosphorylation by regulating the posttranslational modification of protein phosphatase 2A. The pro-memory action of loganin may be attributed to AChE and BChE inhibition [99]. However, the importance of loganin in the treatment of AD is strongly related to suppression of APP processing by inhibition of β-secretase and α-secretase. In addition, loganin reduces inflammation by downregulating iNOS expression and lessening levels of pro-inflammatory mediators, such as TNF-α, NF-κB, and COX-2. This effect is mediated by activating of ERK/MAPK, p38, and JNK signaling pathways [98].

Paeoniflorin, a major monoterpene glycoside in the root of *Paeonia lactiflora*, revealed activity against Aβ cytotoxicity in two in vitro AD models [98]. This iridoid also displayed antioxidant, anti-inflammatory, and anti-apoptotic properties. In vitro experiments revealed that paeoniflorin increased GSH (glutathione—reduced form) capacity and decreased malondialdehyde and NO levels. This antioxidant action is supposed to be related to the activation of Akt/GSK-3β signaling pathway. Furthermore, research saw that the anti-inflammatory effect of paeoniflorin was manifested by reducing the level of NF-κB, TNF-α, IL-1, IL-6. In addition, it was found to inhibit apoptosis by enhancing Bcl-2, while reducing Bax and caspase-3 expression. Finally, this iridoid was seen to prevent mitochondrial dysfunction, restore mitochondrial membrane potential, and maintain Ca^2+^ homeostasis [98,99].

### 3.4. Secoiridoids in the Treatment of AD

Oleuropein and oleuropein aglycone, major constituents of olive oil, were discovered to inhibit the aggregation of tau proteins. In a variety of animal AD models, dietary supplementation of oleuropein aglycone significantly improved the cognitive performance and reduced Aβ deposition in the cortical and hippocampal neurons. The latter effect was due to enhanced clearance of Aβ deposits mediated by upregulating autophagy-related genes and proteins, such as beclin-1, LC3II, p62, and cathepsin B. Oleuropein aglycone-induced autophagy seems to be closely associated with AMPK kinase activation. In turn, reduction of the brain Aβ loads may be due to decreased expression of glutaminyl cyclase (a key enzyme for production of Aβ42 protein), and increased expression of α-secretase and MMP-9 (a metalloproteinase exhibiting α-secretase-like activity). In the rat brain, oleuropein was found to reduce microglia activation and decrease the level of inflammation. Moreover, oleuropein aglycone increased the lifespan of *Caenorhabditis elegans* and reduced ROS levels, increasing SOD activity [106,124].

Oleocanthal, a seco-iridoid dialdehydic-type aglycone, significantly inhibited the tau fibrillization by binding with the lysine amino group of tau protein. In a mouse AD model, oleocanthal increased clearance of Aβ40 from the brain through enhancing the levels of the two main transporting proteins at the BBB—P-gp and LRP-1, and the Aβ degrading proteins—IDE, NEP, apoE, and ABCA-1. Furthermore, oleocanthal reduced Aβ-induced inflammatory responses in the astrocytes, hence, decreasing the levels of IL-1β and GFAP in mice brain tissues. This seco-iridoid also reversed GFAP-induced Aβ deposition and decreased the activity of glutamine and the glucose transporters (GLT-1, GLUT-1)—the proteins that can strengthen synaptic dysfunction. While the Aβ-induced toxicity leads to a decrease in the levels of synaptic neurotransmitter release proteins PSD-95 and SNAP-25, oleocanthal normalized their concentrations, ameliorating synaptic function. Available evidence indicates that dietary supplementation of extra virgin olive oil (25-50 mL daily) could be sufficient to improve the Aβ clearance, relevant in both prevention and treatment of AD [106,125].

## 4. Memory and Learning Improvement

Problems with memory and learning are the most characteristic features of AD and dementia. Such symptoms can be observed in the first stage of AD development and in the progress of dementia and leads to memory loss that disrupts daily life. Another early symptom of AD is the difficulty remembering newly learned information. The following are the most important studies results (in vitro, in silico, and in vivo) of the application of EOs containing monoterpenes in AChE and BuChE inhibition, as well as in countering Aβ accumulation—both having influence on memory/learning dysfunction.

### 4.1. In Vitro and In Silico Studies Results

Our previous studies performed towards understanding AChE inhibitory activity explored the monoterpenes. Such work involved the use of Ellman’s and Marston’s assays, as well as molecular docking [126]. Attention was focused on the following eighteen compounds: isopulegol, (**h**), menthol, (**k**), (**x**), (**y**), (**f**), linalool (**a**), carvone, (**g**), citronellal, terpinene-4-ol, citral (**b**), (**p**), menthone, farnesene, α-phellandrene, and β-myrcene (**e**) (Figure 2). Out of all the terpenes, (**b**), citronellal, carvone, and (**p**) (0.01 mg in Marston’s assay) and (**p**), carvone, menthone, (**f**) and (3.5 mM in Ellman’s assay) demonstrated the highest activity. Molecular docking results obtained for the selected active monoterpenes also revealed significant interactions with amino acids residues of AChE (Ser203, His447, Trp86, Tyr133, Tyr337, and Phe338).

In other work, a rich group of bicyclic monoterpenoids was assessed with regard to AChE inhibition [127]. Such compounds can be divided in terms of skeleton into four groups: pinane, carane, fenchane, and camphene (**t**). Colorimetric Ellman’s methods allowed determining percentage ability to inhibit enzyme activity. Herein, (**i**) (48.5%), fenchol (37.7%), (−)-trans-myrtenol (37.4%), (+)-trans-myrtenol (37.1%), and fenchone (28.2%) showed the highest activity.

In the above research, detailed analysis of structure–activity relationship indicated that hydrocarbon compounds are better inhibitors than alcohols and ketones. Thus, it can be considered that oxygenated moieties decrease inhibitory activity similarly to presence of terminal olefin, whereas compounds with an allylic methyl group show higher inhibitory activity. Considering the aforementioned dependencies, the different activities of similar compounds can be explained. For example, regarding fenchone and fenchol, the first is a weaker inhibitor, therefore the ketone group is less active than alcohol moiety.

Another property that could be useful in countering neurodegeneration is BACE1 inhibition. Marumato et al. performed BACE1 inhibitory analysis of 80 types of aroma compounds, including the monoterpenes [128]. Of these, geranyl acetate, citronellyl acetate, 2-carene, 3-carene, sabinene (**j**), camphor (**n**), and fenchone demonstrated high activity. However, most of the sesquiterpenes revealed weaker activity than the monoterpenes. Inhibition of BACE1 directly impacts upon Aβ formation and aggregation, which in turn influence brain functioning. Besides the aforementioned monoterpenes, in both in vitro and in vivo models, p-cymene (**k**) was another compound that was tested towards Aβ fibrillation [129]. In vitro assay based on observation of fibril formation with use of an electron microscope confirmed the suspicion that (**k**) prevents fibrillation while simultaneously protecting against senile plaque formation. The positive effect of the monoterpenes was observed in animal models through a display of memory enhancement (see: subsection In vivo studies—Monoterpenes).

Similarly to various other diseases, neurodegeneration is closely connected with the oxidative stress and cell death initiated by a high level of free radicals. Oxytosis is a non-apoptotic form of cell death associated with increased oxidative stress and mitochondrial dysfunction. The term was firstly described for oxidative cell death in neuronal cells [130]. The process is initiated by compounds such as glutamate that inhibit the cysteine/glutamate antiporter system.

A significant biological activity is the ability to scavenge free radicals and to provide cell defense against oxidation. A majority of natural compounds reveal high activity in this regard, but in most cases, the mechanism underlying the effect in the cell is not fully understood. Linalool (**a**) is a good example of an active monoterpene holding antioxidant ability (indeed, comparable to vitamin E and lipoic acid) and the ability to prevent lipid oxidation (studies based on unsaturated fatty acids extracted from guinea pig brains) [131].

The mechanism of the antioxidant effect of (**a**) was revealed through studies based on the influence of the monoterpene on physiological function of mitochondria and its potential neuroprotective properties in conditions of glutamate-induced oxytosis in HT-22 cells and in ex vivo organotypic hippocampal slices [130]. The obtained results explicitly indicated that the neuroprotective effect of (**a**) is created by means of mechanisms involving an increase in mitochondrial respiration. Additional studies involving hippocampal slices noted that the monoterpene mediated protection against NMDA-induced excitotoxicity. Thus, it can be said that (**a**) holds a neuroprotective effect that results from restoring mitochondrial function, along with improving mitochondrial morphology, membrane potential, and respiration.

### 4.2. In Vivo Studies Results

Linalool (**a**) (Figure 2), a well-known monoterpene, was also investigated for its ability to mitigate neurodegeneration and to improve cognitive function. In this case, researchers focused on the phospholipid alteration in the brain that leads to neurodegeneration progression and cognitive impairment. In in vivo experiments based on old triple transgenic Alzheimer’s mice (**a**) was administered orally for a three months, after which time behavioral tests were conducted [102]. The undertaken behavioral tests demonstrated cognitive improvement. This effect was found to be due to reduction in extracellular β-amyloidosis, tauopathy, astrogliosis and microgliosis and reduction of pro-inflammatory markers p38 MAPK, NOS2, COX2, and IL-1β. Reverse results were obtained by Coelho et al., who studied the impact of (**a**) on short- and long-term memories through three tests: object recognition task, inhibitory avoidance test and habituation to a novel environment [132]. In this case, a rat model (male Wistar rats) was used and the substance was administered intraperitoneally (i.p.). (**a**) administration revealed various influences on memory in the object recognition task and the new environment recognition test. (**a**) brought about impairment of long-term memory, whereas the effect was not observed for short-term memory. A negative influence was also observed for both memory types in the inhibitory avoidance task in tests using male Wistar rats. This effect is believed to be the outcome of an antagonist capacity as regards NMDA glutaminergic receptors, since another glutamate antagonist also seems to affect memory [133].

Citral (**b**) is an important and common open chain monoterpene (Figure 2). It is a constituent of numerous plant-derived EOs, including lemon grass and verbena. Similarly to other monoterpenes, (**b**) is characterized by holding various biological activities such as anxiogenic and sedative qualities [134]. The cognitive effects of (**b**) on spatial memory were investigated using the Morris water maze test in a male SD rat model. The obtained results revealed an interesting dependency between (**b**) activity and dosage, namely, as indicated by behavioral tests, small doses of the monoterpene (0.1 mg/kg) are able to improve the learning capability of rats, as well as memory, whereas high doses of (**b**) (1.0 mg/kg) can lead to repression of spatial learning and memory in rats [135]. The mechanism of action is explained by the influence of (**b**) on retinoic acid, which has impact upon a vital function in the development of spatial memory in the rat brain.

Limonene (**c**) is another interesting monoterpene (Figure 2). This can be found in the *Rutaceae* family. The monoterpene has various biological effects, including neuroprotective, anti-diabetic, anti-inflammatory, and antioxidant [136,137]. Its activity against neurodegeneration symptoms, namely memory impairment and hippocampal damage, was investigated by in vivo testing, i.e., the Morris water maze and elevated plus-maze tests. Oral administration of (**c**) was found to bring about significantly prevention of weakness of memory and learning and to mitigate neuronal loss due to exposure to stress in the hippocampal region of the rat (male Wistars rats) brain. Limonene’s probable mechanism of action is based on its antioxidant and anti-inflammatory activities [138].

Researchers who focused on the negative influence of high fat diet (HFD) (suspected of increasing the risk of AD) investigated the administration of the monoterpene phenol, thymol (**d**) (Figure 2). Therein, the HFD rat model of AD was used in order to assess the mitigating effect of (**d**), along with its influence on spatial memory improvement. The outcome of this was that detailed analysis revealed that the HFD-fed male Wistars rat model with Aβ impairment positively responded to thymol treatment (observed as spatial memory and learning improvement in the Morris water maze test and passive avoidance test), along with significantly decreased Aβ plaque deposits in the hippocampus [139]. Fang et al. (2017) also studied issues of dependency between HFD and neurodegeneration effects, along with the influence of thymol [140]. Similarly to the previous, these researchers employed in vivo assays, but this time in a male C_57_BL/6J mice model fed with HFD. The tests showed significant improvement of cognitive functions (Morris water maze test), along with decreasing Aβ depositions and hptau in the hippocampus. The mechanism of thymol action is probably based on the antioxidant and anti-inflammatory activities of the compound. Additionally, thymol is able to down-regulate the P-Ser307 IRS-1 level, leading to enhancement of the expression of P-Ser473 AKT and P-Ser9 GSK3β. Researchers also indicated that the up-regulation of nuclear respiratory factor (Nrf2)/heme oxygenase-1(HO-1) pathway is entwined with the protective effects of thymol on cognitive impairments [140,141].

Seifi-Nahavandi et al. (2020) assessed the activity of p-cymene (**k**) against Aβ formation [129]. The research, preceded by in vitro tests, was based on a rat model modified by injection of Aβ1-42 to hippocampus to create the model of AD. Studies were performed for six experimental groups of animals wherein doses of (**k**) were equal to 50 and 100 mg/kg. Results analysis demonstrates that both doses positively influenced learning and memory functions (behavioral tests) and reduce amyloid plaque deposition.

Paeoniflorin (Figure 2) is another monoterpene derivative that displays various biological activities. This was isolated from the dry root of Paeonia. The pinane monoterpene glycoside is characterized by an anti-depressant-like effect and improvement in cognitive ability. The last was studied by way of the Aβ-treated rats model of hippocampal dysfunction as induced by intrahippocampal injection of Aβ [142]. Chronic treatment (20 days) with the monoterpene significantly improved cognitive functions as evaluated by means of the Morris water maze test. According to the research conclusions, the mechanism of paeoniflorin action is through (Ca^2+^) homeostasis, increase of reduced glutathione content, decrease of carbonyl protein (CP) and melanodialdehyde levels, as well as by suppression of NOS activity and NO level.

Lan et al. (2013) performed similar studies confirming the activity of paeoniflorin using adult male Sprague-Dawley rats [143]. The rats were modified by Aβ1-42 injection into the dorsal hippocampus. In these, the paeniflorin (**o**) was administered by injection for 20 days, and cognitive (spatial learning and memory ability) improvement was observed in the Morris water maze test. Additionally, AChE and ChAT activities in the hippocampus were evaluated. Detailed analysis revealed that when given, the monoterpene is able to mitigate spatial learning and memory deficits. Moreover, it can regulate nerve growth factor (NGF)-related signaling, hence strengthening cholinergic functions in the hippocampus [143]). With regard to the probable mechanism of neurodegeneration, this activity is extremely important. It is known that NGF may boost the sprouting of cholinergic neurons and simultaneously rescue the cholinergic neuron from lesion-induced atrophy, therefore enhancing neuronal survival [144,145]. The monoterpene is also able to quickly penetrate through the BBB.

An additional information about positive influence of selected monoterpenes on memory and learning enhancement observed in in vivo models have been presented in Table 1.

## 5. Anxiolytic Effects

Generalized anxiety disorder (GAD) is characterized by excessive, high unrealistic and permanent worry with associated secondary psychic and somatic symptoms. GAD was found to be more chronic than most other psychiatric conditions and often coexists with other psychiatric conditions [152]. Long-term anxiety can be triggered by both somatic (e.g., muscle tension) and psychic symptoms (e.g., sleep disturbance, difficulty in concentrating, tension) [153]. GAD can lead to considerable personal, family, and social impairment [154].

Intensified anxiety is related to increased Aβ presence in healthy, older adults and adults with mild cognitive impairment and AD. It is associated with reductions in memory and related aspects of cognition [155]. The frequency of anxiety among patients with AD varies from 25% to 75% [156]. According to the conclusions of a study of patients with AD [157], the presence of GAD is associated with greater levels of depression, tearfulness, tension, irritability, overt aggression, and mania. Anxiety can adversely affect the progression of AD [158]. Furthermore, increasing evidence has suggested that trait anxiety (a form of state anxiety), one major component of neuroticism predicting individual vulnerability in response to stress, is a risk factor for AD and may correlate with various AD pathologies [158].

Currently, therapy of anxiety includes utilizing psychological approaches, as well as administering anxiolytic drugs such as benzodiazepines, antidepressants, anticonvulsants, and others [153]. EOs constitute a potential new treatment category for anxiety [159].

Research on animal models has demonstrated the beneficial anxiolytic properties found in certain EOs. The method of administration is, however, important for the obtained effects. The most common routes of administration include inhalation, oral and topical approaches [160]. The inhalation of EOs can lead to inducing anxiolytic effects through the CNS—by lung absorption and bloodstream transport, or in stimulating the olfactory system—by bringing about secondary activation of brain regions [161]. Researchers are, however, only beginning to clarify the mechanism behind the anxiolytic-like effect of EOs. Currently, the thought is that the influence of EOs on the CNS is combined with interactions with serotonin receptors, the GABAergic system, voltage-gated Na^+^ channels [159,160], and voltage dependent calcium channels [162]. In terms of anxiolytic-like properties, *Lavendula angustifolia* oil is presumably the best-studied and the most commonly applied EO [160,163].

*Lavandula angustifolia* is a shrub of the *Lamiaceae* family [164]. Lavender is a plant traditionally used as anxiolytic, anticonvulsant, analgesic, sedative and hypnotic [165]. It is grown commercially in the regions of the Mediterranean Sea, in southern Europe, Bulgaria, Russia and the USA. Lavender inflorescences grow in spikes and are crowded with 6–10 pale violet flowers. It grows most optimally in full sun with wind protection. The material used for EO production consists of fresh or dried tops of flowering plants. EO is present in amounts from 2% to 3% and includes over 300 chemical compounds [164]. The compounds that are most abundant in lavender EO are linalool (**a**) (9.3–68.8%) and linalyl acetate (1.2–59.4%). Other important indicated compounds are borneol, α-terpineol (**r**), terpinene-4-ol, and lavandulol acetate. The main group of compounds consist of oxygenated monoterpens (>70%), with the greatest fraction being of monoterpene alcohols [166]. Silexan is a preparation containing 80 mg lavender oil in gelatine capsules for oral use and is derived from the fresh flowers of the plant by steam distillation [167]. The content of active compounds is standardized and contains 36% linalool (**a**) and 34% linalyl acetate [162]. In Germany, Silexan is registered for the treatment of restlessness related to anxious mood [167].

Research into the anxiolytic-like effect of lavender and other EOs containing monoterpenes are shown in Table 2. Herein, anxiolytic-like activity in animal models of compounds belonging to the group of monoterpenes and derivatives, such as carvacrol (**v**) [168], carvacryl acetate [169], (-)-myrtenol [170], (+)-limonene epoxide [170], linalool [102], linalool oxide [171], 1,4-cineole (**v**) [172], (**h**) [173], (**e**), (**b**) [134], citronellol [174], and nerol [175] are listed (Figure 2).

## 6. Insomnia

In developed societies, insomnia is a social problem that is mainly associated with stress, as well as with numerous diseases and the side effects of various drugs. The symptom is also characteristic for neurodegeneration, but in this case, it is exhibited in the form of somnolence. Available data show that sleep disturbances are closely associated with AD diagnosis and with impairment of cognitive functions at long-term follow-up. Research has also indicated an explicit correlation between the accumulation of Aβ and sleep impairment.

Therapy based on CBT-I (Cognitive Behavioral Therapy for Insomnia), while effective in countering insomnia, has not been assessed in terms of cognitive function improvement resulting from Aβ reduction [195]. Data analysis, however, explicitly indicates a correlation between slow wave sleep (SWS) and Aβ accumulation, thus it is thought that increasing SWS reduces amyloid plaques formation [196]. Studies performed by Xie et al. [197] probably provide confirmation of this, as they demonstrated that amyloid plaques in mice cortex were removed more efficiently during the sleep period.

Enhanced tau protein levels and NFTs are common to both AD and sleep disturbance. Indeed, studies that were of 3 months endurance and were based on transgenic mice models housed in normal conditions indicate that increasing the level of phosphorylated tau protein brings about sleep disturbances that are long-lasting and irreversible [198].

Inflammation observed in the brain, being a characteristic feature resulting from neurodegenerative damage, provides confirmation of the correlation between neurodegeneration and insomnia, as inflammation is regulated by cytokines, the level of which can be changed by altering sleep patterns. Available data suggest that insomnia is also associated with higher levels of CRP and IL-6 [199]. Therefore, treatment countering sleep disturbances can significantly improve the cognitive functions impaired because of neurodegeneration. With regard to this, research has noted that numerous plant derivatives show pro-sleeping activity that simultaneously impacts upon neurodegeneration. Among these, several monoterpenes and EOs have revealing the aforementioned activity in in vivo models.

### Monoterpenes

Monoterpenes are responsible for the hypnotic, sedative, and anxiolytic activities noted in several plant-based extracts and EOs. Linalool (**a**) is a monoterpene that has been subject to experimentation. This substance has been evaluated for the purpose of seeing its potential for regulating sleep in experiments using Wistar rats and Kunming mice. These were injected i.p. with pentobarbital sodium [200]. The monoterpene dosage was 400 mg/kg (mice) and 300 mg/kg (rats). During the experiment, sleep rate, sleep latency, and sleep duration were determined. Detailed analysis indicated significant higher (60% in rats, 70% in mice) sleep rate, along with shortened sleep latency and prolonged duration, in comparison to animal groups treated with lavender or lavender oil, as well as controls.

Yang et al. [201] investigated the mechanism behind the hypnotic effect of α-pinene (**h**). The studies, based on in vivo sleep behavior, in silico molecular modeling, and ex vivo examination, revealed that the sleep enhancing property of the monoterpene is brought about by way of direct binding to GABA_A_-benzodiazepine receptors through it acting as a partial modulator at the BZD binding site. The conducted behavioral studies, based on C57BL/6N mice, involved a pentobarbital-induced sleep test that was followed by detailed analysis of sleep architecture. The oral administration of (**h**) induced sedative-hypnotic effects, along with decreasing sleep latency and increasing sleep duration. There was significant difference observed in the influence of the monoterpenes in sleep in NREMS and REMS stages of sleep when compared to controls.

The monoterpene, 3-carene, was found to have similar mechanism of action. This substance is a bicyclic monoterpene, and is the main component of pine tree EO [202]. The hypnotic activity of the compound was examined using C57BL/6N mice and involved a pentobarbital-induced sleep test. 3-carene was administrated orally followed by i.p. injection of pentobarbital. Results analysis explicitly noted the sleep-enhancing activity of the monoterpene. This was ascertained to be the response to 3-carene being a positive modulator of GABA_A_-BZD receptor, confirmation of which was established through molecular docking (3-carene binds to BDZ site of α1 and γ2 subunits of GABA_A_-BZD). The researchers concluded that the compound has potential as a hypnotic drug in pharmacological therapy.

Sleep regulatory activity was also determined for 1,8-cineole by way of experiments utilizing sodium pentobarbital- or ethyl ether-induced assays based on female Swiss mice treated with 50 mg/kg i.p. of the monoterpene [203]). Detailed results analyses of both tests bring to light the positive influence of eucalyptol (**w**) on increasing sleep time. The study group explained the mechanism as being the eucalyptol-mediated modulation of glutamatergic and dopaminergic neurotransmission.

Beyond the aforementioned, the (R)-(−)- and (S)-(+)- carvone enantiomers were assessed with respect to their use in countering insomnia [204]. As with the previous monoterpene, research utilized Swiss mice and pentobarbital-induced hypnosis. The study group administered the compounds i.p. at doses 50, 100, and 200 mg/kg. In the case of both enantiomers, active doses turned out to be 100 and 200 mg/kg. Furthermore, test results indicated that (R)-(−)- carvone was more effective than (S)-(+)- carvone at 100 mg/kg, whereas it was less active at 200 mg/kg in comparison to (+)-enantiomer. The positive effect of administration of these substances, according to the researchers, is associated with regulation of sleep or inhibition of pentobarbital metabolism and potentiation of the GABA_A_ receptor-mediated response.

## 7. Outline of Polyphenols and Essential Oils Rich in Monoterpenes and Polyphenols Revealing Neuroprotective Effect

As mentioned previously, monoterpenes are the main components of various plants usually used in pharmacy, medicine, as well as the food industry. Traditional medicine uses the valuable plants based on their sedative, anti-inflammatory, anti-analgesic, antibacterial, and a lot more biological activities. It is known that essential oils of all plants contain secondary plant metabolites belonging to various groups such as terpenes, polyphenols, glucosides, saponins, etc. Among the most often studied secondary plant metabolites are polyphenols which anti-neurodegenerative activities were confirmed in numerous studies. An important feature of the substances is their high biological activities which can be heightened by synergistic effects [205,206,207]. Due to the fact that polyphenols and monoterpenes are often presented as the most promising natural compounds used in neurodegenerative disorders, the following section will be focused on the aforementioned subject.

### 7.1. In Vitro Studies Results

Plants and their derived essential oils (EOs) are an inexhaustible source of natural active substances that mitigate the effects of illness and disorders. Hence, secondary plant metabolites are subject to constant pharmacological research. Among the substances, gallic acid is one of the most detailed studied polyphenols. Beside high antioxidant activity, its inhibitory activity was confirmed. Studies performed by Jayamani and Shanmugam [208] were focused on inhibitory activity of the compounds against insulin amyloid fibril formation. The in vitro studies based on atomic force microscopy (AFM), Thioflavin T assay, circular dichroism, and Fourier transform-infrared spectroscopy revealed inhibition of insulin amyloid fibrils. The phenomenon is explained by inhibition of conformational transition of α-helix to β-sheet. Additionally, authors suggest that the polyphenol can interact with native insulin leading to prevention of nuclei formation. The valuable activity was also presented by Yu et al. [209] who decided to evaluate ability of gallic acid to prevention Aβ aggregation in both in vitro and in vivo conditions. Similarly to previously presented studies, the scientists decided to use AFM, thioflavin T fluorescence densitometry analyses as well as dynamic light scattering. The study’s results confirmed that the polyphenol can significantly reduce Aβ_1-42_ mediated intracellular calcium influx and neurotoxicity. In order to explain mechanism of action, molecular docking was performed. Detailed analysis revealed that 3,4,5-hydroxyle groups of gallic acid were responsible for stabilization the polyphenol binding to the Lys28-Ala42 salt bridge. The scientists also confirmed the activity in in vivo model [see: Section 7.2. In Vivo Studies Results].

Another important polyphenol that is a promising anti-neurodegenerative agent is epigallocatechin gallate (EGCG). In accordance with available studies results, the compounds is able to inhibit calcitonin, amyloid-β, and α-synuclein amyloid formation,, as well as inhibit hIAPP amyloidogenesis in vitro [210,211,212]. In vitro and in vivo studies were also performed by Franko et al. [213] who confirmed activity of EGDG towards inhibition of amyloid fibril formation. Scientists explained mechanism of EGCG action by simultaneous impact of the following factors: Pi-Pi stacking, Van der Waals, alkyl, Pi-alkyl, conventional hydrogen bonds, and carbon hydrogen bonds interactions between residue and ligand. An activity against senile plaques formation of the polyphenol was studied numerous times. In most cases, EGCG revealed surprisingly high activity. A good example is ability of the polyphenol to bind transthyretin (TTR), that are protein aggregation diseases associated with peripheral neuropathy [214]. In vitro and ex vivo studies confirmed ability of EGCG to inhibit TTR amyloid fibril formation. It is worth to mention about high stability of the molecules interactions as well as the fact that ligand bind strongly to the protein under the studied conditions even though the occupancy obtained was very low. It is known that the valuable compound is able to inhibit aggregation amyloid proteins by impact on α-synuclein and prion protein PrP [215].

It is known that gallic acid and EGCG are not the only active anti-neurodegenerative polyphenols. Among them are substances which revealed high activity in in vitro and in vivo studies. One of the important steps in this type of study that can lead to a more advanced evaluation of compound activity is mechanism of action and structure–activity relationship. Taking into account various structures and moieties of polyphenols, the compounds can bind and interact with various enzymes, amino acid residues, and other types of biomolecules responsible for neurodegeneration including Aβ aggregation, neurofibrillary tangles, neuroinflammatory, etc. An example can be curcumin and nordihydroguaiaretic acid (NDGA) which are able to bind to TTR WT or TTR V30M both in vitro and ex vivo at the T4 binding site. Notice that the acid is characterized by the highest T4 displacement potency and NDGA and curcumin can present different modes of interaction at the T4 binding channel [216]. It is worth to mention that both compounds are able to destabilize Aβ and αS pre-formed fibrils [217], curcumin can disaggregate amyloid fibrils, whereas NDGA decrease levels and size of TTR fibrils [218].

Polyphenols, similarly to monoterpenes, are main constituents of numerous plant essential oils. The mixtures were analyzed towards anti-neurodegenerative activity along with detailed analysis of their component responsible for the biological feature. Ghajarbeygi et al. [219], for example, focused on *Pistacia khinjuk*. Chromatographic analysis revealed that the main compounds of its EO are α-pinene (**h**) and phellandrene (**m**)—known monoterpenes along with phenolic compounds (53.19 mg gallic acid equivalent/PKEO g) evaluated by Azimi et al. [220]. In vitro studies towards AChE and BuChE inhibition, based on Ellman’s method, uncovered their high activity (83,6% and 81,4%, respectively).

Another interesting study was performed on the effects of mixed essential oils (*Citrus aurantii dulcis*, *Citrus lemon*, *Lavandula angustifolia*, *Salvia officinalis*, and *Rosmarinus officinalis*). These were found to incorporate terpenes ((**c**), (**f**), (**e**), (**i**), (**h**), (**j**), (**a**), cymene, and (**l**)) (Figure 2). Besides the terpenes, phenolic compounds constitute important part of the essential oils: caffeic acid, chlorogenic acid, or rosmarinic acid [221,222]. In this case, molecular docking experiments (BuChE) and Ellman’s method (AChE, BuChE) were employed. Docking simulations indicated that (**c**) was the most promising inhibitor, but the mix of essential oils revealed satisfactory activity towards both AChE and BuChE enzymes in Ellman’s assay [223] [Boiangiu et al. 2020].

Armijos et al. [224] researched the BuChE inhibitory activity of essential oils from the traditional Ecuadorian beverage, horchata lojana. The tea-like mixture consists of more than 20 medicinal and aromatic herbs and flowers. Among these are *Amaranthus cruentus* L., *Matricaria recutita* L., *Foeniculum vulgare* Mill., *Borago officinalis* L., *Sambucus nigra* L., *Dianthus caryophyllus* L., *Pelargonium graveolens* L’Her. ex Aiton., *Pelargonium odoratissimum* (L.), *Equisetum bogotense* Kunth., *Melissa officinalis* L., *Mentha piperita* L., *Ocimum basilicum* L., *Malva arborea* (L.), *Alcea rosea* (L.), *Fuchsia magellanica* Lam. and a few more. It is known that the plants and their essential oils have numerous pro-health activities, including anti-inflammatory, hepatic, diuretic, tonic, analgesic, relaxant, digestive, and anti-tumor effects [225,226]. Chemical analysis of essential oil compositions indicated neral, geranial, carvone, myrcene, nerol and γ-muurolene as the most important ingredients. All essential oils obtained from various compositions of horchata, however, revealed AChE inhibitory activity with IC50 above 250 µg/mL, hence they can be labeled as inactive inhibitors. In contrast, BuChE inhibition was much higher, therefore, consumption of the tea-like mixture can slightly improve memory [224].

Describing high biological activity of essential oils, synergism and antagonism of their components should be underlined. It is known that most of the compounds can interact with another which influences on their activity. It can be said about positive (synergism) and negative (antagonism) effects which are commonly used in medicine and pharmacy. Modern therapy is often based on mixtures of drugs both natural and synthetic [227]. The effects are very important to explain mode of action of herbal medicine. An important example, used in neurodegeneration, is *Panax ginseng* and *Ginko biloba* for which stronger activity was observed for their combination as compared to the single treatments [228]. Synergism was also observed for *Hypericum* constituents (hyperforin, hypercines, amentoflavone, rutin, hyperosid, xanthones, amethoflavon) which beneficial antidepressant action was linked with cooperation of the aforementioned compounds [229]. It is highly probable that anti-neurodegenerative activity of essential oils rich in monoterpenes and polyphenols results from synergy of main components. In most cases in vitro studies results reveal lower activity of single components that whole essential oils and the differences are confirmed by calculations based on synergy definition [230].

### 7.2. In Vivo Studies Results

In vivo models are a bridge between in vitro and clinical studies. As mentioned in previous subsection, polyphenols are promising secondary plant metabolites in neurodegeneration treatment. This activity was confirmed in in vivo models. An example can be studies Yu et al. [209] who confirmed in vitro activity in in vivo model. The studies, based on APPswe/PS1dE9 transgenic mice (C57BL/6 black-ground express chimeric amyloid precursor protein encoding the Swedish mutations), revealed that oral administration of gallic acid significantly improved spatial reference memory, spatial working memory, and reduced more severe deficits. Additionally, hippocampal long-term-potentiation was improved with increased expression of synaptic marker proteins. High activity against Aβ aggregation along with explanation of mechanism of action was presented by Rezai-Zadeh et al. [231]. In this case, the scientists focused on epigallocatechin-3-gallate and it influence on reduction of Aβ generation in Swedish mutant APP-overexpressing mise (Tg APP_sw_line 2576). The studied substance was administered intraperitoneally for 60 days. Based on obtained results, high activity of EGCG was observed. The activity was explained by cleavage promotion of α-C-terminal fragment of APP and elevation of N-terminal APP cleavage product, soluble APP-α. The decrease of Aβ levels and plaques are closely linked with promotion of nonamyloidogenic α-secretase proteolytic pathway. Multi-directional activity of EGCG against neurodegeneration was also evaluated by Ferreira et al. [232]. Detailed analysis of in vivo studies (transgenic mice for human TTR V30M) results along with semiquantitative immunohistochemistry and Western blot analysis of mice tissues showed that the polyphenol inhibits TTR toxic aggregates deposition in about 50% along the gastrointestinal tract and peripheral nervous system. Treatment based on the natural compound also caused decrease of non-fibrillar TTR deposition and disaggregation of amyloid deposits. Additionally, reduction of amyloid deposition markers and serum amyloid P component was observed.

Similarly to in vitro studies, in vivo studies based on essential oils rich in both monoterpenes and polyphenols were conducted. It is worth to mention about Ghajarbeygi et al. [219], who examined the activity of *Pistacia khinjuk* essential oil. As aforementioned in previous section, the plant contains various class of compounds including monoterpenes and phenols phenolic compounds (53.19 mg gallic acid equivalent/PKEO g). This was shown to have high inhibitory activity (AChE and BuChE) in in vitro assay. These researchers employed a rat model (adult male Wistar rats) treated with Aβ peptide in order to induce amnesia. Additionally, the inhibitory potential (AChE/BuChE) of the oil was compared with Donepezil. Ghajarbeygi et al. chose to orally administer *Pistacia khinjuk* EO at concentrations of 1, 2 and 3%. The obtained results demonstrated the activity of this EO towards decreasing AChE and BuChE activity. This outcome was higher for EO 3%.

Inhalation turned out to be an effective way of administering essential oil in a rat model performed by Bagci et al. [233]. These researchers focused their attention on *Anthriscus nemorosa* essential oil and its activity in preventing memory impairment in scopolamine-treated rats. The plant, commonly known as ‘beaked chervil’, belongs to the *Apiaceae* family and is used in traditional medicine in Turkey. GC-MS/GC-FID analysis indicated the presence of the following secondary metabolites in the essential oil of the plant: caryophyllene, germacrene, β-elemene, α-terpineol (**r**) being major compounds, whereas the monoterpenes are represented by α-pinene (**h**), sabinene (**j**), β-pinene (**i**), β-phellandrene (**m**), β-ocimene (**p**), trans-pinocarveol (**q**), 3-cyclohexen-1-ol (**z**), α-terpineol (**r**)**,** and pregeijerene (**s**) (summary 19.40%) (Figure 2). Phenolic contents was confirmed by additional studies (74.6 mg GAE.L-1) [234]. Inhalation with the essential oil (1% and 3% for 21 days) demonstrated a positive influence on memory and on other symptoms of neurodegeneration (anxiety and depression). This suggests that inhalation with *Anthriscus nemorosa* can be considered as a promising way to prevent memory impairment.

Lavender is a crucial example of a plant demonstrating pro-sleeping effects. This is observed for intranasal uses of essential oils in form of aromatherapy. Gas chromatography analysis has revealed the following compounds to be the main constituents of Lavender EO: linalyl acetate, linalool (**a**), terpinene-4-ol, 1,8-cineole, camphor (**n**), limonene (**c**), α-terpineol (**r**)**,** and lavendulyl acetate (Figure 2), but simultaneously phenolic compounds were identified: 2-O-glucosilcoumaric acid, o-coumaric acid, rosmarinic acid, apigenin-7-O-glucoside, coumarin, herniarin, luteolin, and apigenin [235].

With regard to lavender EO, a possible mechanism having influence on sleep quality is reduction of salivary cortisol levels, this in turn leads to lessening of chronic stress. It should be underlined that sleep quality induced by lavender EO is closely related with its anxiolytic properties. These are connected with induced NMDA receptor antagonism and inhibition of the serotonin transporter (SERT) [236]. According to Ko et al. [237], lavender’s essential oils constituents are able to bind to the glutamate N-methyl-D-aspartate receptors and serotonin transporters.

The effect of lavender aromatherapy was also examined toward its influence on blood melatonin levels in 60-years-old of both sexes [238]. The studies were based on inhalation of lavender essential oil (2.95 mg) for 30 min (eight sessions), for four weeks. Evaluation of blood melatonin levels after the experiment showed increase in the level of the hormone in all participants. Moreover, such effects brought with it simultaneous sleep quality improvement.

Similar study results were also obtained for *Tetraclinis articulate*, a plant used in traditional medicine as an antibacterial, antifungal, anti-inflammatory, and antioxidant agent. Sadiki et al. [239] investigated the effect of the plant’s essential oil on memory and brain oxidative stress in an amyloid-β peptide-induced Alzheimer’s disease amyloidosis model. Similarly to previous studies, these researchers employed a rat model, along with inhalation administration of essential oil at doses of 1% and 3% for 21 days. The experiment demonstrated the positive effect of inhalation towards memory improvement in Y-maze and radial arm maze testing. The outcome was assigned to the contained EO constituent monoterpenes (monoterpene hydrocarbons—47.08%, oxygenated monoterpenes—41.59%): (**h**), (**t**), (**e**), (**c**), (**n**) and bornyl acetate (**u**) (Figure 2) but simultaneously the presence of polyphenols (caffeic acid and quercetin’s derivatives) significantly improve their biological activities [240].

## 8. Conclusions

Monoterpenes and their derivatives constitute a very interesting and attractive treatment category for AD and related disorders, like cognitive disorders, insomnia, and anxiety. Such various effects can be due to their unusual multi-targeted mechanism of action on the CNS. Numerous monoterpene components exhibited anxiolytic properties in a variety of animal models, including elevated plus maze, open field, and marble burying tests. These actions are believed to be realized through activation of GABA_A_/BDZ receptors, enhancement of serotonergic and inhibition of glutamatergic neurotransmission. The same mechanisms seem to be involved in hypnotic effects of monoterpenes. However, their pro-memory action, widely revealed in the Morris water maze, novel object recognition, and passive avoidance tests, is closely related to AChE inhibition. Interestingly, even a weak or moderate effect on AChE does not exclude monoterpenes from their therapeutic use. Such substances may serve as template structures to boost activity of existing antidementive medications. An example is galantamine. Monoterpenoids can offer, however, much more than just pro-memory properties. Among them, iridoids and secoiridoids showed beneficial potential in different in vitro and in vivo models of AD. These active substances inhibited β-secretase, decreasing Aβ protein production, and enhance Aβ protein clearance from the brain. Additional anti-inflammatory and antioxidative actions increase usefulness of monoterpenoids in the treatment of neurodegenerative diseases. The route of administration often determines the drug usefulness in clinical practice. Monoterpenes and monoterpenoids showed their activity not only after parenteral, but also oral application. The inhalation route also seems very attractive. Larger clinical trials should be conducted to confirm the place of monoterpenes and monoterpenoids in the treatment of AD.

## Figures and Tables

**Table 1:** In vivo studies towards memory and learning improvement by monoterpenes.

Monoterpene	In Vivo Model	Type of Test	Administration of Compound	Observations	Possible Mechanism of Action	References
thymol	Wistar Rats impaired with intrahippocampal injection of amyloid or intraperitoneal injection of scopolamine	Morris water maze task	intraperitoneally; 0.5, 1, or 2 mg/kg	Improvement cognitive functions; reversed negative effect of Aβ and scopolamine	Antioxidant and anti-inflammatory activity of thymol	[146]
carvacrol	Wistar Rats impaired with intrahippocampal injection of amyloid or intraperineal injection of scopolamine	Morris water maze task	intraperitoneally; 0.5, 1, or 2 mg/kg	Improvement cognitive functions; reversed negative effect of Aβ and scopolamine	Antioxidant and anti-inflammatory activity of carvacrol	[146]
	Sprague-Dawley rats with memory impairment and neuroinflammation gained by lipopolysaccharide injected into the lateral ventricle	Object recognition task; Morris water maze test	Intraperitoneally; 25 mg/kg, 100 mg/kg	Memory improvement; significantly attenuation of induced defects in cognitive functions	Attenuation of expression of inflammatory factors in the brain (interleukin-1β, taumor necrosis factor-α, cyclooxygenase-2). Increasing expression of brain-derived neurotropic factor mRNA and decrease expression of Toll-like receptor 4 mRNA.	[147]
	Male Wistar rats with memory impairment obtained by lead acetate drinking water	Morris water maze test	Orally; 25,50, and 100 mg/kg	Improvement of memory and learning	Decreasing lipid peroxidation and oxidation stress by improvement of activity of antioxidant enzymes (SOD, CAT, GPx)	[148]
linalool	C57BL/6J mice model anesthetized and stereotypically injected aggregated *Aβ* into hippocampus	Morris water maze test; passive avoidance test	intraperitoneally; 100 mg/kg	Cognitive improvement effect without negative influence on locomotor activity	Decreasing of apoptosis and oxidative stress induced by A*β* depending on Nrf2/HO-1 pathway	[104]
	Homozygous triple transgenic AD model (3xTg-AD) and nontransgenic (Non-Tg) mice	Morris water maze test	Orally, 25 mg/kg	reversion of cognitive and emotional deficits	antioxidant activity; decreasing of *β*-amyloid level	[102]
limonene	WKY and SHRsp rats with ischemia induced by occlusion of right middle cerebral artery—evaluation of influence of limonene on memory after stroke	Morris water maze test; novel object recognition test	Intraperitoneally; 20 mg/kg	protection against memory impairment	antioxidant effects through increasing SOD and CAT activities, decreasing MDA levels, increasing GSH levels and decreasing ROS levels.	[149]
α-pinene	scopolamine-induced memory deficit in C57BL/6 mice	Morris water maze test; passive avoidance test	Intraperitoneally; 10 mg/kg	memory enhancing activity and learning improvement	regulation of expression of proteins related to synthesis of acetylcholine and antioxidant defense system	[150]
α-terpinene	female Wistar rats	inhibitory avoidance task;	Orally; 0.5, 0.75 and 1.0 mL/kg	memory impairment	altering activity of enzymes responsible for neuronal plasticity and hydrolysis of ADP and ATP	[151]

**Table 2:** Studies of monoterpenes in animal and human models of anxiety.

Essential Oils and Main Chemical Compounds	Method of Administration	Dosage	In Vivo Model	Anxiety Model	Observed Effect, Main Results	Reference
*α*-pinene, camphene, *β*-pinene, *β*-phellandrene (*Abies sachalinensis*)	i.p. Inhalation	0.6 g/kg body weight 3.6 mg/l air	Mouse	Elevated plus-maze test (EPM)	i.p. administration did not produce an anxiolytic-like effect, albeit, a significant anxiolytic-like effect was observed after inhalation. The influence of the way of administration on the distribution of the main compounds was then analyzed. The anxiolytical effect is suspected to be due to the achievement of adequate levels in the brain after inhalation	[176]
fragranol, fragranyl acetate, *γ*-terpinene, thymol, eugenol (*Achillea umbellata*)	p.o.	50, 100 and 150 mg/kg	Mouse	Light/Dark transition test	The applied EO showed antianxiety properties (increased time spent in the light compartment and decreased time spent in the dark in a dose-dependent manner), but these are very likely signs of intoxication and not of possible beneficial effects of the plant volatiles.	[177]
p-cymene, 1,8-cineole, terpinen-4-ol, limonene (*Alpinia zerumbet*)	Inhalation	0.087 ppm, 0.87 ppm and 8.7 ppm for 90 min	Mouse	Elevated plus-maze test	0.087 and 8.7 ppm of EO obviously showed anxiolytic-like activity.	[178]
limonene, *β*-pinene, *β*-myrcene, sabinene (*Citrus aurantium*)	p.o.	1, 5, 10, 50 mg/kg for 30 min or 14-day repeated (1, 5, 10 mg/kg/day)	Mouse	Light/Dark Box Test	The anxiolytic-like activity observed after acute (5 mg/kg) or 14-day repeated (1 mg/kg/day) dosing was mediated by the serotonergic system (5-HT_1A_ receptors). The obtained results suggest that the EO does not function through the GABA-benzodiazepine receptor complex	[179]
limonene, *β*-pinene, *β*-myrcene, sabinene (*Citrus aurantium)*	p.o.	0.5 and 1.0 g/kg	Mouse	Elevated plus-maze test	EPM results suggest the anxiolytic effect of the EO.	[180]
limonene, *β*-pinene, *β*-myrcene, sabinene (*Citrus aurantium*)	p.o.	Acutely pretreated at 1, 5, 10 or 50 mg/kg and 1, 5 or 10 mg/kg/day for 14 days	Mouse	Light/Dark Box test	The EO possesses a significant anxiolytic-like effect, and the present results strongly suggest the involvement of 5- HT1A-receptors.	[179]
limonene, *β*-pinene, *β*-myrcene, sabinene (*Citrus aurantiu*)	Inhalation	1.0%, 2.5% and 5.0% for 7 min	Rat	Open-field test behavioral Social interaction test Elevated plus-maze test	The concentration of 2.5% increased both the time of the animals spent in the open arms of the EPM and the time of social interactions for rats. This was longer than that of the diazepam group (1.5 mg/kg *i.p*) and testifies to an anxiolytic-like effect. Results suggest a possible central action of EO	[181]
limonene, geranyl acetate and trans-limonene-oxide (*Citrus limon*)	p.o.	50, 100 and 150 mg/kg/day for 30 days	Mouse	Elevated plus-maze test	Dosage of EO was able to significantly increase the time of permanence and the number of entrances in the open arms, indicating a positive response. The study suggests anxiolytic effects of the used EO that might involve an action on benzodiazepine-type receptors.	[182]
limonene, geranyl acetate and trans-limonene-oxide (*Citrus limon*)	Inhalation	150 µL once a day for 2 weeks	Rat	Elevated plus-maze test	Long-term EO exposure (both male and female rats) increased anxiety in the plus-maze test, i.e., it reduced the time spent in the open arms. The obtained results indicate an anxiogenic-like effect.	[183]
limonene, myrcene (*Citrus sinensis*)	Inhalation	100, 200, 400 μL for 5 min	Rat	Elevated plus-maze test Light/Dark Box Test	All the used doses of the EO showed anxiolytic effects. To exclude the possibility that this result was due to non-specific effects of odor exposure, the rats’ response to *Melaleuca alternifolia* EO was also assessed.	[184]
sabinene, 1,8-cineole, limonene, myrcene (*Lantana camara*)	Inhalation	0.0004 and 0.04 mg for 60 min	Mouse	Open-field test	The administered EO significantly decreased locomotor activity in a dose-dependent manner. The reduction of locomotor activity may indicate anxiolytic effects.	[185]
linalol, linalyl acetate, terpinene-4-ol, ocimene, cineole (*Lavandula angustifolia*)	Inhalation	0.5%, 2.5%, 5.0% for 15 min	Mouse	Marble-burying test	The result of the study suggests that the perception of smells is not crucial for inducing the anxiolytic-like effect in normal and anosmic mice.	[161]
linalol, linalyl acetate, terpinene-4-ol, ocimene, cineole (*Lavandula angustifolia*)	Inhalation	4 µL/L air for 90 min	Mouse	Elevated plus-maze test	A similar anxiolytic-like effect was noticed in stressed and non-stressed mice	[186]
linalol, linalyl acetate, terpinene-4-ol, ocimene, cineole (*Lavandula angustifolia*)	Inhalation	1–5% for 15 min	Mouse	Marble-burying test Elevated plus-maze test	The anxiolytic-like effect of lavender oil may be mediated by serotonergic transmission, and the participation of the GABAA/BDZ complex is unlikely. Administering the oil by inhalation induced rats to reduce the number of marbles buried. This occurred at a dose that did not affect locomotor activity. 5% lavender EO attenuated the serotonin syndrome induced by 40 mg/kg fluoxetine plus 80 mg/kg 5-hydroxytryptophan.	[187]
linalol, linalyl acetate, terpinene-4-ol, ocimene, cineole (*Lavandula angustifolia*)	Inhalation	Vaporized oil for 90 min	Mouse	Elevated plus-maze test	Olfactory stimulation is not necessarily required for lavender EO to show an anxiolytic action. An increase in hippocampal serotonin turn-over after lavender inhalation was, however, noticed. This enhanced the proposal of a serotonergic mediation of the anxiolytic-like effects of EO.	[188]
linalol, linalyl acetate, terpinene-4-ol, ocimene, cineole (*Lavandula angustifolia*)	Inhalation	0.1–1.0 mL for 30 min or 1 h	Rat	Open-field test	An anxiolytic-like effect was established. Higher doses had some effects on behavior similar to those of 10 mg/kg body weight chlordiazepoxide (CDP). Doses 0.5 mL–1.0 mL increased immobility. A sedative effect can also occur at the upper doses.	[189]
linalol, linalyl acetate, terpinene-4-ol, ocimene, cineole (*Lavandula angustifolia*)	Inhalation	Refreshed three times daily with four drops of the EO by 24 h and 14 days	Gerbil	Elevated plus-maze test	Lavender EO inhalations have an anxiolytic profile in gerbils that is similar to that of the anxiolytic diazepam (1 mg/kg). Sex differences in protected head-dip (an ethological indicator of anxiety) was indicated–females showed a significant decrease in response to this test. Two-week exposure to the scent of lavender increased exploratory behavior in female gerbils, indicating a further decrease in anxiety in this sex.	[190]
linalol, linalyl acetate, terpinene-4-ol, ocimene, cineole (*Lavandula angustifolia*)	Inhalation	1 mL of 10% EO	Sheep	Reaction to stress (isolation)	Genetic differences in temperament determined whether lavender oil alleviates or exacerbates the behavioral and/or endocrine correlates of anxiety in sheep. Exposure of calm sheep to lavender oil i.a. decreased the agitation score and the plasma concentrations of cortisol prior to isolation, compared to control, and thus they exhibited anxiolytic-like effects. The parameters of the above-mentioned tests carried out on nervous sheep were opposite, lavender oil showed an anxiogenic effect.	[191]
linalol, linalyl acetate, terpinene-4-ol, ocimene, cineole (*Lavandula angustifolia*)	p.o.	1–30 mg/kg/day for 3 days	Mouse	Elevated plus-maze test	Anxiolytic-like effect was verified because the test mice showed an increase in open arms exploration in the elevated plus-maze compared to positive control (diazepam and pregablin). The results of the experiment suggest non-selective inhibition of voltage operated calcium channels as a mechanism of anxiolytic effect.	[162]
linalol, linalyl acetate, terpinene-4-ol, ocimene, cineole (*Lavandula angustifolia*)	p.o.	6-week-intake 80 mg/day	Human (adults with GAD)	Hamilton Anxiety Rating Scale (HAMA) total score, Double-blind, double-dummy, randomized, reference-controlled multicentre trial	The results suggest that silexan effectively ameliorates generalized anxiety at a comparable degree to that of lorazepam (0.5 mg/day) administration.	[192]
linalol, linalyl acetate, terpinene-4-ol, ocimene, cineole (*Lavandula angustifolia*)	p.o.	10-weeks-intake 80 mg/day	Human	Hamilton Anxiety Scale (HAMA), Double-blind, randomized, placebo-controlled multicentre trial	An anxiolytic effect was established. Symptoms of anxiety were reduced more explicitly in patients taking Silexan than that of those in the placebo group. Additionally, there was an improvement in the patients’ mental condition.	[167]
geranial, neral, d-limonene, *β*-myrcene, *α*-terpinyl acetate (*Litsea cubeba*)	p.o.	100, 300 and 500 mg/kg/day for 7 days	Mouse	Elevated plus-maze test	An anxiolytic effect was established. *L. cubeba* oil has a potent effect on the central nervous system of mice.	[193]
thymol, *γ*-terpinene, trans-caryophyllene, *β*-selinene, p-cymene (*Ocimum gratissimum*)	Inhalation	4.0 × 10^−10^ −4.0 × 10^−1^ mg for 60 min	Mouse	Light/Dark transition test	Inhalation of EO (at a concentration of 4.0 × 10^−4^ mg) showed anxiolytic potential and did not cause any deleterious effects on motor coordination.	[194]

i.p.—intraperitoneally; p.o.—per os.

## Data Availability

Not applicable.

## Abbreviations

Aβ	amyloid β
ABCA-1	ATP-binding cassette transporter-A1
ACh	acetylcholine
AChE	acetylcholinesterase
AD	Alzheimer’s disease
AICD	intracellular domain CTFβ
AMPK	AMP-activated protein kinase
ApoE	apolipoprotein E
APP	amyloid β precursor protein
BACE1	β-secretase 1
BBB	blood-brain barrier
BChE	butyrylcholinesterase
BDNF	brain-derived neurotrophic factor
ChAT	choline acetyltrasferase
COX	cyclooxygenase
ECE	endothelin converting enzymes
ER	endoplasmic reticulum
ERK	extracellular signal–regulated kinase
CREB	cAMP-response element binding protein
GAD	generalized anxiety disorder
GAP-43	growth associated protein 43
GFAP	glial fibrillary acidic protein
GLP-1R	glucagon-like peptide-1 receptor
GLT-1	glutamine transporter-1
GLUT-1	glucose transporter-1
GPx	glutathione peroxidase
GSHPx	glutathione peroxidase
GSH-ST	glutathione S-transferase
HFO	hogh fat diet
hptau	hyperphosphorylated tau protein
IDE	insulin degrading enzyme
IκB	nuclear factor of kappa light polypeptide gene enhancer in B-cells inhibitor
IL-1	interleukin-1
JNK	c-Jun N-terminal kinase
LC3II	lysosomal cysteine protease 3II
LRP-1	lipoprotein receptor-related protein 1
mAChR	muscarinic acetylcholine receptors
MAPK	mitogen-activated protein kinase
mTOR	mammalian target of rapamycin
nAChR	nicotinic acetylcholine receptors
NEP	neprilysin
NF-κB	nuclear factor kappa light chain enhancer of activated B cells
NFTs	neurofibrillary tangles
NGF	nerve growth factor
NMDA	N-methyl-D-aspartic acid
NOS	nitric oxide synthase
PGE	prostaglandin
PHF	paired helical filaments
PKC	protein kinase C
PS	presenilin
PSD-95	postsynaptic density protein 95
PTEN	phosphatase and tensin homolog
RAGE	receptor for advanced glycation endproducts
ROS	reactive oxygen species
SERT	serotonin transporter
SNAP-25	synaptosomal nerve-associated protein-25
SOD	superoxide dismutase
SWS	slow wave sleep
TACE	α-secretase
TNF	tumor necrozing factor
TrkB	tropomyosin receptor kinase B

## References

[1] Wojtunik-Kulesza K., Oniszczuk A., Waksmundzka-Hajnos M. (2019). An attempt to elucidate the role of iron and zinc ions in development of Alzheimer’s and Parkinson’s diseases. Biomed. Pharmacother..

[2] Kulshreshtha A., Piplani P. (2016). Current pharmacotherapy and putative disease-modifying therapy for Alzheimer’s disease. Neurol. Sci. Off. J. Ital. Neurol. Soc. Ital. Soc. Clin. Neurophysiol..

[3] Blackburn L., Achor S., Allen B., Bauchmire N., Dunnington D., Klisovic R.B., Naber S.J., Roblee K., Samczak A., Tomlinson-Pinkham K. (2017). The effect of aromatherapy on insomnia and other common symptoms among patients with acute leukemia. Oncol. Nurs. Forum.

[4] Da Silva G.L., Luft C., Lunardelli A., Amaral R.H., da Silva Melo D.A., Donadio M.V.F., Nunes F.B., de Azambuja M.S., Santana J.C., Moraes C.M.B. (2015). Antioxidant, analgesic and anti-inflammatory effects of lavender essential oil. An. Acad. Bras. Cienc..

[5] Popova A., Dalemska Z., Mihaylova D., Hristova I., Alexieva I. (2016). *Melissa officinalis* L.- GC Profile and Antioxidant Activity. Int. J. Pharmacogn. Phytochem. Res..

[6] Wojtunik-Kulesza K.A., Kasprzak K., Oniszczuk T., Oniszczuk A. (2019). Natural Monoterpenes: Much More than Only a Scent. Chem. Biodivers..

[7] Maggio A., Rosselli S., Bruno M. (2016). Essential oils and pure volatile compounds as potential drugs in Alzheimer’s disease therapy: An updated review of the literature. Curr. Pharm. Des..

[8] Adorjan B., Buchbauer G. (2010). Biological properties of essential oils: An updated review. Flavour Fragr. J..

[9] Chen J., Jiang Q.-D., Chai Y.-P., Zhang H., Peng P., Yang X.-X. (2016). Natural terpenes as penetration enhancers for transdermal drug delivery. Molecules.

[10] Wippold F.J., Cairns N., Vo K., Holtzman D.M., Morris J.C. (2008). Neuropathology for the neuroradiologist: Plaques and tangles. Am. J. Neuroradiol..

[11] Yankner B.A., Caceres A., Duffy L.K. (1990). Nerve growth factor potentiates the neurotoxicity of β amyloid. Proc. Natl. Acad. Sci. USA.

[12] Hardy J., Selkoe D.J. (2002). The amyloid hypothesis of Alzheimer’s disease: Progress and problems on the road to therapeutics. Science.

[13] Duce J.A., Tsatsanis A., Cater M.A., James S.A., Robb E., Wikhe K., Leong S.L., Perez K., Johanssen T., Greenough M.A. (2010). Iron-export ferroxidase activity of β-amyloid precursor protein is inhibited by zinc in Alzheimer’s disease. Cell.

[14] Priller C., Bauer T., Mitteregger G., Krebs B., Kretzschmar H.A., Herms J. (2006). Synapse formation and function is modulated by the amyloid precursor protein. J. Neurosci. Off. J. Soc. Neurosci..

[15] Isbert S., Wagner K., Eggert S., Schweitzer A., Multhaup G., Weggen S., Kins S., Pietrzik C.U. (2012). APP dimer formation is initiated in the endoplasmic reticulum and differs between APP isoforms. Cell. Mol. Life Sci..

[16] Abeysinghe G., Kuchira M., Kudo G., Masuo S., Ninomiya A., Takahashi K., Utad A.S., Hagiwara D., Nomura N., Takaya N. (2020). Fungal mycelia and bacterial thiamine establish a mutualistic growth mechanism. Life Sci. Alliance.

[17] Godyń J., Jończyk J., Panek D., Malawska B. (2016). Therapeutic strategies for Alzheimer’s disease in clinical trials. Pharmacol. Rep..

[18] Wang D.-S., Dickson D.W., Malter J.S. (2006). beta-Amyloid degradation and Alzheimer’s disease. J. Biomed. Biotechnol..

[19] Winkler E., Kamp F., Scheuring J., Ebke A., Fukumori A., Steiner H. (2012). Generation of Alzheimer disease-associated amyloid β42/43 peptide by γ-secretase can be inhibited directly by modulation of membrane thickness. J. Biol. Chem..

[20] Lewczuk P., Kamrowski-Kruck H., Peters O., Heuser I., Jessen F., Popp J., Bürger K., Hampel H., Frölich L., Wolf S. (2010). Soluble amyloid precursor proteins in the cerebrospinal fluid as novel potential biomarkers of Alzheimer’s disease: A multicenter study. Mol. Psychiatry.

[21] Rabbito A., Dulewicz M., Kulczyńska-Przybik A., Mroczko B. (2020). Biochemical Markers in Alzheimer’s Disease. Int. J. Mol. Sci..

[22] Haass C., Lemere C.A., Capell A., Citron M., Seubert P., Schenk D., Lannfelt L., Selkoe D.J. (1995). The Swedish mutation causes early-onset Alzheimer’s disease by beta-secretase cleavage within the secretory pathway. Nat. Med..

[23] Leong Y.Q., Ng K.Y., Chye S.M., Ling A.P.K., Koh R.Y. (2020). Mechanisms of action of amyloid-beta and its precursor protein in neuronal cell death. Metab. Brain Dis..

[24] Nikolaev A., McLaughlin T., O’Leary D.D.M., Tessier-Lavigne M. (2009). APP binds DR6 to trigger axon pruning and neuron death via distinct caspases. Nature.

[25] Sennvik K., Bogdanovic N., Volkmann I., Fastbom J., Benedikz E. (2004). Beta-secretase-cleaved amyloid precursor protein in Alzheimer brain: A morphologic study. J. Cell. Mol. Med..

[26] Fraser P.E., Yang D.S., Yu G., Lévesque L., Nishimura M., Arawaka S., Serpell L.C., Rogaeva E., St George-Hyslop P. (2000). Presenilin structure, function and role in Alzheimer disease. Biochim. Biophys. Acta.

[27] Suh Y.-H., Checler F. (2002). Amyloid precursor protein, presenilins, and alpha-synuclein: Molecular pathogenesis and pharmacological applications in Alzheimer’s disease. Pharmacol. Rev..

[28] Octave J.N., Essalmani R., Tasiaux B., Menager J., Czech C., Mercken L. (2000). The role of presenilin-1 in the gamma-secretase cleavage of the amyloid precursor protein of Alzheimer’s disease. J. Biol. Chem..

[29] Selkoe D.J. (2001). Presenilin, Notch, and the genesis and treatment of Alzheimer’s disease. Proc. Natl. Acad. Sci. USA.

[30] Morley J.E., Farr S.A., Banks W.A., Johnson S.N., Yamada K.A., Xu L. (2010). A physiological role for amyloid-beta protein: Enhancement of learning and memory. J. Alzheimer’s Dis..

[31] Puzzo D., Privitera L., Leznik E., Fà M., Staniszewski A., Palmeri A., Arancio O. (2008). Picomolar amyloid-beta positively modulates synaptic plasticity and memory in hippocampus. J. Neurosci. Off. J. Soc. Neurosci..

[32] Chen G., Xu T., Yan Y., Zhou Y., Jiang Y., Melcher K., Xu H.E. (2017). Amyloid beta: Structure, biology and structure-based therapeutic development. Acta Pharmacol. Sinica..

[33] Walsh D.M., Lomakin A., Benedek G.B., Condron M.M., Teplow D.B. (1997). Amyloid β-protein fibrillogenesis: Detection of a protofibrillar intermediate. J. Biol. Chem..

[34] Lesné S.E., Sherman M.A., Grant M., Kuskowski M., Schneider J.A., Bennett D.A., Ashe K.H. (2013). Brain amyloid-β oligomers in ageing and Alzheimer’s disease. Brain.

[35] Ezeani M., Omabe M. (2015). A New Perspective of Lysosomal Cation Channel-Dependent Homeostasis in Alzheimer’s Disease. Mol. Neurobiol..

[36] Mark R.J., Hensley K., Butterfield D.A., Mattson M.P. (1995). Amyloid beta-peptide impairs ion-motive ATPase activities: Evidence for a role in loss of neuronal Ca2+ homeostasis and cell death. J. Neurosci. Off. J. Soc. Neurosci..

[37] Varadarajan S., Yatin S., Aksenova M., Butterfield D.A. (2000). Review: Alzheimer’s amyloid beta-peptide-associated free radical oxidative stress and neurotoxicity. J. Struct. Biol..

[38] Heneka M.T., Golenbock D.T., Latz E. (2015). Innate immunity in Alzheimer’s disease. Nat. Immunol..

[39] Neniskyte U., Neher J.J., Brown G.C. (2011). Neuronal death induced by nanomolar amyloid β is mediated by primary phagocytosis of neurons by microglia. J. Biol. Chem..

[40] Weggen S., Eriksen J.L., Das P., Sagi S.A., Wang R., Pietrzik C.U., Findlay K.A., Smith T.E., Murphy M.P., Bulter T. (2001). A subset of NSAIDs lower amyloidogenic Abeta42 independently of cyclooxygenase activity. Nature.

[41] Devi L., Prabhu B.M., Galati D.F., Avadhani N.G., Anandatheerthavarada H.K. (2006). Accumulation of Amyloid Precursor Protein in the Mitochondrial Import Channels of Human Alzheimer’s Disease Brain Is Associated with Mitochondrial Dysfunction. J. Neurosci..

[42] Abdallah C.G., Averill L.A., Krystal J.H., Southwick S.M., Arnsten A.F.T. (2016). Glutamate and Norepinephrine Interaction: Relevance to Higher Cognitive Operations and Psychopathology. Behav. Brain Sci..

[43] Canevari L., Abramov A.Y., Duchen M.R. (2004). Toxicity of amyloid beta peptide: Tales of calcium, mitochondria, and oxidative stress. Neurochem. Res..

[44] Chiarini A., Dal Pra I., Whitfield J.F., Armato U. (2006). The killing of neurons by beta-amyloid peptides, prions, and pro-inflammatory cytokines. Ital. J. Anat. Embryol. Arch. Ital. Anat. Ed. Embriol..

[45] Iwata N., Higuchi M., Saido T.C. (2005). Metabolism of amyloid-beta peptide and Alzheimer’s disease. Pharmacol. Ther..

[46] Eckman E.A., Reed D.K., Eckman C.B. (2001). Degradation of the Alzheimer’s amyloid beta peptide by endothelin-converting enzyme. J. Biol. Chem..

[47] Bertram L., Blacker D., Mullin K., Keeney D., Jones J., Basu S., Yhu S., McInnis M.G., Go R.C., Vekrellis K. (2000). Evidence for genetic linkage of Alzheimer’s disease to chromosome 10q. Science.

[48] Tucker H.M., Kihiko M., Caldwell J.N., Wright S., Kawarabayashi T., Price D., Walker D., Scheff S., McGillis J.P., Rydel R.E. (2000). The plasmin system is induced by and degrades amyloid-beta aggregates. J. Neurosci. Off. J. Soc. Neurosci..

[49] Backstrom J.R., Lim G.P., Cullen M.J., Tökés Z.A. (1996). Matrix metalloproteinase-9 (MMP-9) is synthesized in neurons of the human hippocampus and is capable of degrading the amyloid-beta peptide (1-40). J. Neurosci. Off. J. Soc. Neurosci..

[50] Hu J., Igarashi A., Kamata M., Nakagawa H. (2001). Angiotensin-converting enzyme degrades Alzheimer amyloid beta-peptide (A beta); retards A beta aggregation, deposition, fibril formation; and inhibits cytotoxicity. J. Biol. Chem..

[51] McDermott J.R., Gibson A.M. (1996). Degradation of Alzheimer’s beta-amyloid protein by human cathepsin D. Neuroreport.

[52] Deane R., Wu Z., Sagare A., Davis J., Du Yan S., Hamm K., Xu F., Parisi M., LaRue B., Hu H.W. (2004). LRP/amyloid beta-peptide interaction mediates differential brain efflux of Abeta isoforms. Neuron.

[53] Deane R., Du Yan S., Submamaryan R.K., LaRue B., Jovanovic S., Hogg E., Welch D., Manness L., Lin C., Yu J. (2003). RAGE mediates amyloid-beta peptide transport across the blood-brain barrier and accumulation in brain. Nat. Med..

[54] Shibata M., Yamada S., Kumar S.R., Calero M., Bading J., Frangione B., Holtzman D.M., Miller C.A., Strickland D.K., Ghiso J. (2000). Clearance of Alzheimer’s amyloid-ss(1-40) peptide from brain by LDL receptor-related protein-1 at the blood-brain barrier. J. Clin. Investig..

[55] Cirrito J.R., Deane R., Fagan A.M., Spinner M.L., Parsadanian M., Finn M.B., Jiang H., Prior J.L., Sagare A., Bales K.R. (2005). P-glycoprotein deficiency at the blood-brain barrier increases amyloid-beta deposition in an Alzheimer disease mouse model. J. Clin. Investig..

[56] Zlokovic B.V., Deane R., Sallstrom J., Chow N., Miano J.M. (2005). Neurovascular pathways and Alzheimer amyloid beta-peptide. Brain Pathol. Zurich Switz..

[57] Mattson M.P. (2004). Pathways Towards and Away from Alzheimer’s Disease. Nature.

[58] Miyakawa T., Kimura T., Hirata S., Fujise N., Ono T., Ishizuka K., Nakabayashi J. (2000). Role of blood vessels in producing pathological changes in the brain with Alzheimer’s disease. Ann. N. Y. Acad. Sci..

[59] Cuchillo-Ibanez I., Seereeram A., Byers H.L., Leung K.-Y., Ward M.A., Anderton B.H., Hanger D.P. (2008). Phosphorylation of tau regulates its axonal transport by controlling its binding to kinesin. FASEB J. Off. Publ. Fed. Am. Soc. Exp. Biol..

[60] Dixit R., Ross J.L., Goldman Y.E., Holzbaur E.L.F. (2008). Differential regulation of dynein and kinesin motor proteins by tau. Science.

[61] Huang H.-C., Jiang Z.-F. (2009). Accumulated amyloid-beta peptide and hyperphosphorylated tau protein: Relationship and links in Alzheimer’s disease. J. Alzheimer’s Dis..

[62] Bloom G.S. (2014). Amyloid-β and tau: The trigger and bullet in Alzheimer disease pathogenesis. JAMA Neurol..

[63] Lewczuk P., Lelental N., Lachmann I., Holzer M., Flach K., Brandner S., Engelborghs S., Teunissen C.E., Zetterberg H., Molinuevo J.L. (2017). Non-phosphorylated tau as a potential biomarker of Alzheimer’s Disease: Analytical and diagnostic characterization. J. Alzheimer’s Dis..

[64] Hugon J., Mouton-Liger F., Cognat E., Dumurgier J., Paquet C. (2018). Blood-Based Kinase Assessments in Alzheimer’s Disease. Front. Aging Neurosci..

[65] Yuskaitis C.J., Jope R.S. (2009). Glycogen synthase kinase-3 regulates microglial migration, inflammation, and inflammation-induced neurotoxicity. Cell. Signal..

[66] Lee S., Hall G.F., Shea T.B. (2011). Potentiation of tau aggregation by cdk5 and GSK3β. J. Alzheimer’s Dis..

[67] Srebro Z., Wiliński B., Sura P. (2000). Oxidative stress in Alzheimer’s disease. Folia Med. Cracov..

[68] Kaltschmidt B., Uherek M., Volk B., Baeuerle P.A., Kaltschmidt C. (1997). Transcription factor NF-kappaB is activated in primary neurons by amyloid beta peptides and in neurons surrounding early plaques from patients with Alzheimer disease. Proc. Natl. Acad. Sci. USA.

[69] Salim S. (2017). Oxidative Stress and the Central Nervous System. J. Pharmacol. Exp. Ther..

[70] Akiyama H., Barger S., Barnum S., Bradt B., Bauer J., Cole G.M., Cooper N.R., Eikelenboom P., Emmerling M., Fiebich B.L. (2000). Inflammation and Alzheimer’s disease. Neurobiol. Aging.

[71] Sayre L.M., Smith M.A., Perry G. (2001). Chemistry and biochemistry of oxidative stress in neurodegenerative disease. Curr. Med. Chem..

[72] Morales I., Guzmán-Martínez L., Cerda-Troncoso C., Farías G.A., Maccioni R.B. (2014). Neuroinflammation in the pathogenesis of Alzheimer’s disease. A rational framework for the search of novel therapeutic approaches. Front. Cell. Neurosci..

[73] Calsolaro V., Edison P. (2016). Neuroinflammation in Alzheimer’s disease: Current evidence and future directions. Alzheimer’s Dement. J. Alzheimer’s Assoc..

[74] Domingues C., Cruz e Silva O.A.B., Henriques A.G. (2017). Impact of Cytokines and Chemokines on Alzheimer’s Disease Neuro-pathological Hallmarks. Curr. Alzheimer Res..

[75] Zhao N., Liu C.-C., Qiao W., Bu G. (2018). Apolipoprotein E, Receptors, and Modulation of Alzheimer’s Disease. Biol. Psychiatry.

[76] Butterfield D.A., Mattson M.P. (2020). Apolipoprotein E and oxidative stress in brain with relevance to Alzheimer’s disease. Neurobiol. Dis..

[77] Woolf N.J., Butcher L.L. (2011). Cholinergic systems mediate action from movement to higher consciousness. Behav. Brain Res..

[78] Picciotto M.R., Higley M.J., Mineur Y.S. (2012). Acetylcholine as a neuromodulator: Cholinergic signaling shapes nervous system function and behavior. Neuron.

[79] Bekdash R.A. (2021). The Cholinergic System, the Adrenergic System and the Neuropathology of Alzheimer’s Disease. Int. J. Mol. Sci..

[80] Briggs R., Kennelly S.P., O’Neill D. (2016). Drug treatments in Alzheimer’s disease. Clin. Med..

[81] Ferreira-Vieira T.H., Guimaraes I.M., Silva F.R., Ribeiro F.M. (2016). Alzheimer’s disease: Targeting the Cholinergic System. Curr. Neuropharmacol..

[82] Egea J., Buendia I., Parada E., Navarro E., León R., Lopez M.G. (2015). Anti-inflammatory role of microglial alpha7 nAChRs and its role in neuroprotection. Biochem. Pharmacol..

[83] Wang H.Y., Lee D.H., D’Andrea M.R., Peterson P.A., Shank R.P., Reitz A.B. (2000). beta-Amyloid(1–42) binds to alpha7 nicotinic acetylcholine receptor with high affinity. Implications for Alzheimer’s disease pathology. J. Biol. Chem..

[84] Lombardo S., Maskos U. (2015). Role of the nicotinic acetylcholine receptor in Alzheimer’s disease pathology and treatment. Neuropharmacology.

[85] Hascup K.N., Hascup E.R. (2016). Soluble Amyloid-β42 Stimulates Glutamate Release through Activation of the α7 Nicotinic Acetylcholine Receptor. J. Alzheimer’s Dis..

[86] Jiang S., Li Y., Zhang C., Zhao Y., Bu G., Xu H., Zhang Y.-W. (2014). M1 muscarinic acetylcholine receptor in Alzheimer’s disease. Neurosci. Bull..

[87] Caccamo A., Oddo S., Billings L.M., Green K.N., Martinez-Coria H., Fisher A., LaFerla F.M. (2006). M1 receptors play a central role in modulating AD-like pathology in transgenic mice. Neuron.

[88] Bukke V.N., Archana M., Villani R., Romano A.D., Wawrzyniak A., Balawender K., Orkisz S., Beggiato S., Serviddio G., Cassano T. (2020). The Dual Role of Glutamatergic Neurotransmission in Alzheimer’s Disease: From Pathophysiology to Pharmacotherapy. Int. J. Mol. Sci..

[89] Rupsingh R., Borrie M., Smith M., Wells J.L., Bartha R. (2011). Reduced hippocampal glutamate in Alzheimer disease. Neurobiol. Aging.

[90] Jakob-Roetne R., Jacobsen H. (2009). Alzheimer’s disease: From pathology to therapeutic approaches. Angew. Chem. Int. Ed. Engl..

[91] Kothari S., Bala N., Patel A.B., Donovan A., Narayanaswami V. (2021). The LDL receptor binding domain of apolipoprotein E directs the relative orientation of its C-terminal segment in reconstituted nascent HDL. Biochim. Biophys. Acta BBA-Biomembr..

[92] Kim M., Bezprozvanny I. (2021). Differences in Recycling of Apolipoprotein E3 and E4—LDL Receptor Complexes—A Mechanistic Hypothesis. Int. J. Mol. Sci..

[93] Sotolongo K., Ghiso J., Rostagno A. (2020). Nrf2 activation through the PI3K/GSK-3 axis protects neuronal cells from Aβ-mediated oxidative and metabolic damage. Alzheimer’s Res. Ther..

[94] Hamilton K., Harvey J. (2021). The Neuronal Actions of Leptin and the Implications for Treating Alzheimer’s Disease. Pharmaceuticals.

[95] (2021). Protective Effects of Carvacrol Against Diabetes-Induced Reproductive Damage in Male Rats: Modulation of Nrf2/HO-1 Signalling Pathway and Inhibition of Nf-kB-Mediated Testicular Apoptosis and Inflammation. Arkali. Andrologia. Wiley Online Library. https://onlinelibrary.wiley.com/doi/10.1111/and.13899.

[96] Ramos A.G.B., de Menezes I.R.A., da Silva M.S.A., Torres Pessoa R., de Lacerda Neto L.J., Rocha Santos Passos F., Melo Coutinho H.D., Iriti M., Quintans-Júnior L.J. (2020). Antiedematogenic and Anti-Inflammatory Activity of the Monoterpene Isopulegol and Its β-Cyclodextrin (β-CD) Inclusion Complex in Animal Inflammation Models. Foods.

[97] Dinda B., Debnath S., Harigaya Y. (2007). Naturally occurring secoiridoids and bioactivity of naturally occurring iridoids and secoiridoids. A review, part 2. Chem. Pharm. Bull..

[98] Habtemariam S. (2018). Iridoids and Other Monoterpenes in the Alzheimer’s Brain: Recent Development and Future Prospects. Molecules.

[99] Volcho K.P., Laev S.S., Ashraf G.M., Aliev G., Salakhutdinov N.F. (2018). Application of monoterpenoids and their derivatives for treatment of neurodegenerative disorders. Curr. Med. Chem..

[100] Stavrakov G., Philipova I., Zheleva-Dimitrova D., Valkova I., Salamanova E., Konstantinov S., Doytchinova I. (2017). Docking-based design and synthesis of galantamine-camphane hybrids as inhibitors of acetylcholinesterase. Chem. Biol. Drug Des..

[101] Silva Brum L.F., Emanuelli T., Souza D.O., Elisabetsky E. (2001). Effects of linalool on glutamate release and uptake in mouse cortical synaptosomes. Neurochem. Res..

[102] Sabogal-Guáqueta A.M., Osorio E., Cardona-Gómez G.P. (2016). Linalool reverses neuropathological and behavioral impairments in old triple transgenic Alzheimer’s mice. Neuropharmacology.

[103] Zhong Z., Wang B., Dai M., Sun Y., Sun Q., Yang G., Bian L. (2013). Carvacrol alleviates cerebral edema by modulating AQP4 expression after intracerebral hemorrhage in mice. Neurosci. Lett..

[104] Xu P., Wang K., Lu C., Dong L., Gao L., Yan M., Aibai S., Yang Y., Liu X. (2017). Protective effects of linalool against amyloid beta-induced cognitive deficits and damages in mice. Life Sci..

[105] Khan A., Vaibhav K., Javed H., Tabassum R., Ahmed M.E., Khan M.M., Khan M.B., Shrivastava P., Islam F., Siddiqui M.S. (2014). 1,8-cineole (eucalyptol) mitigates inflammation in amyloid Beta toxicated PC12 cells: Relevance to Alzheimer’s disease. Neurochem. Res..

[106] Dinda B., Dinda M., Kulsi G., Chakraborty A., Dinda S. (2019). Therapeutic potentials of plant iridoids in Alzheimer’s and Parkinson’s diseases: A review. Eur. J. Med. Chem..

[107] Liu H.-C., Hong C.-J., Liu C.-Y., Lin K.-N., Tsai S.-J., Liu T.-Y., Chi C.-W., Wang P.-N. (2001). Association analysis of the 5-HT6 receptor polymorphism C267T with depression in patients with Alzheimer’s disease. Psychiatry Clin. Neurosci..

[108] Liu J., He Q.-J., Zou W., Wang H.-X., Bao Y.-M., Liu Y.-X., An L.-J. (2006). Catalpol increases hippocampal neuroplasticity and up-regulates PKC and BDNF in the aged rats. Brain Res..

[109] Wang J.-H., Li W.-T., Yu S.-T., Xie H., Han H.-R. (2014). Catalpol regulates function of hypothalamic-pituitary-adrenocortical-axis in an Alzheimer’s disease rat model. Pharmazie.

[110] Wang Z., Huang X., Zhao P., Zhao L., Wang Z.-Y. (2018). Catalpol Inhibits Amyloid-β Generation Through Promoting α-Cleavage of APP in Swedish Mutant APP Overexpressed N2a Cells. Front. Aging Neurosci..

[111] Li H., Cao L., Ren Y., Jiang Y., Xie W., Li D. (2018). GLP-1 receptor regulates cell growth through regulating IDE expression level in Aβ1-42-treated PC12 cells. Biosci. Rep..

[112] Liu J., Liu Z., Zhang Y., Yin F. (2015). Leptin signaling plays a critical role in the geniposide-induced decrease of tau phosphorylation. Acta Biochim. Biophys. Sin..

[113] Liu J.-H., Yin F., Guo L.-X., Deng X.-H., Hu Y.-H. (2009). Neuroprotection of geniposide against hydrogen peroxide induced PC12 cells injury: Involvement of PI3 kinase signal pathway. Acta Pharmacol. Sin..

[114] Flaherty D.B., Soria J.P., Tomasiewicz H.G., Wood J.G. (2000). Phosphorylation of human tau protein by microtubule-associated kinases: GSK3beta and cdk5 are key participants. J. Neurosci. Res..

[115] Lv C., Liu X., Liu H., Chen T., Zhang W. (2014). Geniposide attenuates mitochondrial dysfunction and memory deficits in APP/PS1 transgenic mice. Curr. Alzheimer Res..

[116] Zhang H., Zhao C., Lv C., Liu X., Du S., Li Z., Wang Y., Zhang W. (2017). Geniposide Alleviates Amyloid-Induced Synaptic Injury by Protecting Axonal Mitochondrial Trafficking. Front. Cell. Neurosci..

[117] Lv C., Wang L., Liu X., Yan S., Yan S.S., Wang Y., Zhang W. (2015). Multi-faced neuroprotective effects of geniposide depending on the RAGE-mediated signaling in an Alzheimer mouse model. Neuropharmacology.

[118] Liu J., Zhang Y., Deng X., Yin F. (2013). Geniposide decreases the level of Aβ1-42 in the hippocampus of streptozotocin-induced diabetic rats. Acta Biochim. Biophys. Sin..

[119] Morrison C.D. (2009). Leptin signaling in brain: A link between nutrition and cognition?. Biochim. Biophys. Acta.

[120] Nam K.N., Choi Y.-S., Jung H.-J., Park G.H., Park J.-M., Moon S.-K., Cho K.-H., Kang C., Kang I., Oh M.S. (2010). Genipin inhibits the inflammatory response of rat brain microglial cells. Int. Immunopharmacol..

[121] Yamazaki M., Sakura N., Chiba K., Mohri T. (2001). Prevention of the neurotoxicity of the amyloid beta protein by genipin. Biol. Pharm. Bull..

[122] Gao C., Liu Y., Jiang Y., Ding J., Li L. (2014). Geniposide ameliorates learning memory deficits, reduces tau phosphorylation and decreases apoptosis via GSK3β pathway in streptozotocin-induced alzheimer rat model. Brain Pathol. Zurich Switz..

[123] Chen C., Zhang H., Xu H., Xue R., Zheng Y., Wu T., Lian Y. (2018). Harpagoside Rescues the Memory Impairments in Chronic Cerebral Hypoperfusion Rats by Inhibiting PTEN Activity. J. Alzheimer’s Dis..

[124] Cordero J.G., García-Escudero R., Avila J., Gargini R., García-Escudero V. (2018). Benefit of Oleuropein Aglycone for Alzheimer’s Disease by Promoting Autophagy. Oxid. Med. Cell. Longev..

[125] Batarseh Y.S., Mohamed L.A., Al Rihani S.B., Mousa Y.M., Siddique A.B., El Sayed K.A., Kaddoumi A. (2017). Oleocanthal ameliorates amyloid-β oligomers’ toxicity on astrocytes and neuronal cells: In vitro studies. Neuroscience.

[126] Wojtunik-Kulesza K.A., Targowska-Duda K., Klimek K., Ginalska G., Jóźwiak K., Waksmundzka-Hajnos M., Cieśla Ł. (2017). Volatile terpenoids as potential drug leads in Alzheimer’s disease. Open Chem..

[127] Miyazawa M., Yamafuji C. (2005). Inhibition of acetylcholinesterase activity by bicyclic monoterpenoids. J. Agric. Food Chem..

[128] Marumoto S., Okuno Y., Miyazawa M. (2017). Inhibition of β-Secretase Activity by Monoterpenes, Sesquiterpenes, and C13 Norisoprenoids. J. Oleo Sci..

[129] Seifi Nahavandi B., Yaghmaei P., Ahmadian S., Ebrahim-Habibi A., Ghobeh M. (2020). Effects of Terpinolene and Physical Activity on Memory and Learning in a Model of Alzheimer’s Disease among Rats. Qom Univ. Med. Sci. J..

[130] Sabogal-Guáqueta A.M., Hobbie F., Keerthi A., Oun A., Kortholt A., Boddeke E., Dolga A. (2019). Linalool attenuates oxidative stress and mitochondrial dysfunction mediated by glutamate and NMDA toxicity. Biomed. Pharmacother..

[131] Celik S., Ozkaya A. (2002). Effects of intraperitoneally administered lipoic acid, vitamin E, and linalool on the level of total lipid and fatty acids in guinea pig brain with oxidative stress induced by H2O2. J. Biochem. Mol. Biol..

[132] Coelho V.R., Gianesini J., Von Borowski R., Mazzardo-Martins L., Martins D.F., Picada J.N., Santos A.R.S., Brum L.F.S., Pereira P. (2011). (−)-Linalool, a naturally occurring monoterpene compound, impairs memory acquisition in the object recognition task, inhibitory avoidance test and habituation to a novel environment in rats. Phytomed. Int. J. Phytother. Phytopharm..

[133] Vianna M.R.M., Izquierdo L.A., Barros D.M., de Souza M.M., Rodrigues C., Sant’Anna M.K., Medina J.H., Izquierdo I. (2001). Pharmacological differences between memory consolidation of habituation to an open field and inhibitory avoidance learning. Braz. J. Med. Biol. Res..

[134] Do Vale T.G., Furtado E.C., Santos J.G., Viana G.S.B. (2002). Central effects of citral, myrcene and limonene, constituents of essential oil chemotypes from Lippia alba (Mill.) n.e. Brown. Phytomed. Int. J. Phytother. Phytopharm..

[135] Yang Z., Xi J., Li J., Qu W. (2009). Biphasic effect of citral, a flavoring and scenting agent, on spatial learning and memory in rats. Pharmacol. Biochem. Behav..

[136] Chaudhary S.C., Siddiqui M.S., Athar M., Alam M.S. (2012). D-Limonene modulates inflammation, oxidative stress and Ras-ERK pathway to inhibit murine skin tumorigenesis. Hum. Exp. Toxicol..

[137] Murali R., Saravanan R. (2012). Antidiabetic effect of d-limonene, a monoterpene in streptozotocin-induced diabetic rats. Biomed. Prev. Nutr..

[138] Bigdeli Y., Asle-Rousta M., Rahnema M. (2019). Effects of Limonene on Chronic Restraint Stress-Induced Memory Impairment and Anxiety in Male Rats. Neurophysiology.

[139] Asadbegi M., Yaghmaei P., Salehi I., Komaki A., Ebrahim-Habibi A. (2017). Investigation of thymol effect on learning and memory impairment induced by intrahippocampal injection of amyloid beta peptide in high fat diet- fed rats. Metab. Brain Dis..

[140] Fang F., Li H., Qin T., Li M., Ma S. (2017). Thymol improves high-fat diet-induced cognitive deficits in mice via ameliorating brain insulin resistance and upregulating NRF2/HO-1 pathway. Metab. Brain Dis..

[141] Hong Y., An Z. (2018). Hesperidin attenuates learning and memory deficits in APP/PS1 mice through activation of Akt/Nrf2 signaling and inhibition of RAGE/NF-κB signaling. Arch. Pharm. Res..

[142] Zhong S.-Z., Ge Q.-H., Li Q., Qu R., Ma S.-P. (2009). Peoniflorin attentuates Aβ(1-42)-mediated neurotoxicity by regulating calcium homeostasis and ameliorating oxidative stress in hippocampus of rats. J. Neurol. Sci..

[143] Lan Z., Chen L., Fu Q., Ji W., Wang S., Liang Z., Qu R., Kong L., Ma S. (2013). Paeoniflorin attenuates amyloid-beta peptide-induced neurotoxicity by ameliorating oxidative stress and regulating the NGF-mediated signaling in rats. Brain Res..

[144] Niewiadomska G., Mietelska-Porowska A., Mazurkiewicz M. (2011). The cholinergic system, nerve growth factor and the cytoskeleton. Behav. Brain Res..

[145] Niewiadomska G., Komorowski S., Baksalerska-Pazera M. (2002). Amelioration of cholinergic neurons dysfunction in aged rats depends on the continuous supply of NGF. Neurobiol. Aging.

[146] Azizi Z., Ebrahimi S., Saadatfar E., Kamalinejad M., Majlessi N. (2012). Cognitive-enhancing activity of thymol and carvacrol in two rat models of dementia. Behav. Pharmacol..

[147] Lee B., Yeom M., Shim I., Lee H., Hahm D.-H. (2020). Inhibitory effect of carvacrol on lipopolysaccharide-induced memory impairment in rats. Korean J. Physiol. Pharmacol..

[148] Zare Mehrjerdi F., Niknazar S., Yadegari M., Akbari F.A., Pirmoradi Z., Khaksari M. (2020). Carvacrol reduces hippocampal cell death and improves learning and memory deficits following lead-induced neurotoxicity via antioxidant activity. Naunyn. Schmiedebergs Arch. Pharmacol..

[149] Wang X., Li G., Shen W. (2018). Protective effects of D-Limonene against transient cerebral ischemia in stroke-prone spontaneously hypertensive rats. Exp. Ther. Med..

[150] Lee G.-Y., Lee C., Park G.H., Jang J.-H. (2017). Amelioration of Scopolamine-Induced Learning and Memory Impairment by α-Pinene in C57BL/6 Mice. Evid. Based Complement. Altern. Med..

[151] Baldissera M.D., Souza C.F., Grando T.H., Sagrillo M.R., De Brum G.F., Nascimento K., Peres D.S., Maciel M.F., Silveira S.O., Da Luz S.C.A. (2016). Memory deficit, toxic effects and activity of Na^+^, K^+^-ATPase and NTPDase in brain of Wistar rats submitted to orally treatment with alpha-terpinene. Environ. Toxicol. Pharmacol..

[152] Haskins J.T. (1999). Generalized anxiety disorder. Epidemiology, impact of comorbidity, and natural history. Postgrad. Med..

[153] Bilkei-Gorzo A., Ströhle A. (2005). Anxiety and Anxiolytic Drugs.

[154] Altunoz U., Kokurcan A., Kirici S., Bastug G., Ozel-Kizil E.T. (2018). Clinical characteristics of generalized anxiety disorder: Older vs. young adults. Nord. J. Psychiatry.

[155] Pietrzak R.H., Lim Y.Y., Neumeister A., Ames D., Ellis K.A., Harrington K., Lautenschlager N.T., Restrepo C., Martins R.N., Masters C.L. (2015). Amyloid-β, Anxiety, and Cognitive Decline in Preclinical Alzheimer Disease: A Multicenter, Prospective Cohort Study. JAMA Psychiatry.

[156] Porter V.R., Buxton W.G., Fairbanks L.A., Strickland T., O’Connor S.M., Rosenberg-Thompson S., Cummings J.L. (2003). Frequency and Characteristics of Anxiety Among Patients with Alzheimer’s Disease and Related Dementias. J. Neuropsychiatry Clin. Neurosci..

[157] Chemerinski E., Petracca G., Manes F., Leiguarda R., Starkstein S.E. (1998). Prevalence and correlates of anxiety in Alzheimer’s disease. Depress. Anxiety.

[158] Li S., Wang C., Wang W., Tan J. (2021). Trait anxiety, a personality risk factor associated with Alzheimer’s Disease. Prog. Neuropsychopharmacol. Biol. Psychiatry.

[159] Wang Z.-J., Heinbockel T. (2018). Essential Oils and Their Constituents Targeting the GABAergic System and Sodium Channels as Treatment of Neurological Diseases. Molecules.

[160] Ramsey J.T., Shropshire B.C., Nagy T.R., Chambers K.D., Li Y., Korach K.S. (2020). Essential Oils and Health. Yale J. Biol. Med..

[161] Chioca L.R., Antunes V.D.C., Ferro M.M., Losso E.M., Andreatini R. (2013). Anosmia does not impair the anxiolytic-like effect of lavender essential oil inhalation in mice. Life Sci..

[162] Schuwald A.M., Nöldner M., Wilmes T., Klugbauer N., Leuner K., Müller W.E. (2013). Lavender oil-potent anxiolytic properties via modulating voltage dependent calcium channels. PLoS ONE.

[163] Takahashi M., Satou T., Ohashi M., Hayashi S., Sadamoto K., Koike K. (2011). Interspecies comparison of chemical composition and anxiolytic-like effects of lavender oils upon inhalation. Nat. Prod. Commun..

[164] Prusinowska R., Śmigielski K.B. (2014). Composition, biological properties and therapeutic effects of lavender (*Lavandula angustifolia* L). A review. Herba Polonica.

[165] Nasiri Lari Z., Hajimonfarednejad M., Riasatian M., Abolhassanzadeh Z., Iraji A., Vojoud M., Heydari M., Shams M. (2020). Efficacy of inhaled Lavandula angustifolia Mill. Essential oil on sleep quality, quality of life and metabolic control in patients with diabetes mellitus type II and insomnia. J. Ethnopharmacol..

[166] Smigielski K., Prusinowska R., Raj A., Sikora M., Woliñska K., Gruska R. (2011). Effect of Drying on the Composition of Essential Oil from Lavandula angustifolia. J. Essent. Oil Bear. Plants.

[167] Kasper S. (2013). An orally administered lavandula oil preparation (Silexan) for anxiety disorder and related conditions: An evidence based review. Int. J. Psychiatry Clin. Pract..

[168] Melo F.H.C., Venâncio E.T., de Sousa D.P., de França Fonteles M.M., de Vasconcelos S.M.M., Viana G.S.B., de Sousa F.C.F. (2010). Anxiolytic-like effect of Carvacrol (5-isopropyl-2-methylphenol) in mice: Involvement with GABAergic transmission. Fundam. Clin. Pharmacol..

[169] Pires L.F., Costa L.M., Silva O.A., de Almeida A.A.C., Cerqueira G.S., de Sousa D.P., de Freitas R.M. (2013). Anxiolytic-like effects of carvacryl acetate, a derivative of carvacrol, in mice. Pharmacol. Biochem. Behav..

[170] Moreira M.R.C., da Silva Stiebbe Salvadori M.G., de Almeida A.A.C., de Sousa D.P., Jordán J., Satyal P., de Freitas R.M., de Almeida R.N. (2014). Anxiolytic-like effects and mechanism of (−)-myrtenol: A monoterpene alcohol. Neurosci. Lett..

[171] Linck V.M., da Silva A.L., Figueiró M., Caramão E.B., Moreno P.R.H., Elisabetsky E. (2010). Effects of inhaled Linalool in anxiety, social interaction and aggressive behavior in mice. Phytomedicine.

[172] Gomes P.B., Feitosa M.L., Silva M.I.G., Noronha E.C., Moura B.A., Venâncio E.T., Rios E.R.V., de Sousa D.P., de Vasconcelos S.M.M., de Franaç Fonteles M.M. (2010). Anxiolytic-like effect of the monoterpene 1,4-cineole in mice. Pharmacol. Biochem. Behav..

[173] Satou T., Kasuya H., Maeda K., Koike K. (2014). Daily inhalation of α-pinene in mice: Effects on behavior and organ accumulation. Phytother. Res..

[174] Umezu T., Ito H., Nagano K., Yamakoshi M., Oouchi H., Sakaniwa M., Morita M. (2002). Anticonflict effects of rose oil and identification of its active constituents. Life Sci..

[175] Marques T., Marques M.L.B.G.C.B., Lima D., Siqueira H., Neto J., Branco M.S.B.G.C., Souza A., Sousa D., Freitas R. (2013). Evaluation of the neuropharmacological properties of nerol in mice. World J. Neurosci..

[176] Satou T., Matsuura M., Takahashi M., Umezu T., Hayashi S., Sadamoto K., Koike K. (2011). Anxiolytic-like effect of essential oil extracted from Abies sachalinensis. Flavour Fragr. J..

[177] Radulović N.S., Dekić M.S., Ranđelović P.J., Stojanović N.M., Zarubica A.R., Stojanović-Radić Z.Z. (2012). Toxic essential oils: Anxiolytic, antinociceptive and antimicrobial properties of the yarrow *Achillea umbellata* Sibth. et Sm. (Asteraceae) volatiles. Food Chem. Toxicol..

[178] Murakami S., Matsuura M., Satou T., Hayashi S., Koike K. (2009). Effects of the Essential Oil from Leaves of *Alpinia zerumbet* on Behavioral Alterations in Mice. Nat. Prod. Commun..

[179] Costa C.A.R.A., Cury T.C., Cassettari B.O., Takahira R.K., Flório J.C., Costa M. (2013). *Citrus aurantium* L. essential oil exhibits anxiolytic-like activity mediated by 5-HT1A-receptors and reduces cholesterol after repeated oral treatment. BMC Complement. Altern. Med..

[180] Carvalho-Freitas M.I.R., Costa M. (2002). Anxiolytic and Sedative Effects of Extracts and Essential Oil from *Citrus aurantium* L. Biol. Pharm. Bull..

[181] Leite M.P., Fassin J., Baziloni E.M.F., Almeida R.N., Mattei R., Leite J.R. (2008). Behavioral effects of essential oil of *Citrus aurantium* L. inhalation in rats. Rev. Bras. Farmacogn..

[182] Lopes Campêlo L.M., Gonçalves e Sá C., de Almeida A.A.C., da Costa J.P., Marques T.H.C., Feitosa C.M., Saldanha G.B., de Freitas R.M. (2011). Sedative, anxiolytic and antidepressant activities of Citrus limon (Burn) essential oil in mice. Pharm..

[183] Ceccarelli I., Lariviere W.R., Fiorenzani P., Sacerdote P., Aloisi A.M. (2004). Effects of long-term exposure of lemon essential oil odor on behavioral, hormonal and neuronal parameters in male and female rats. Brain Res..

[184] Faturi C.B., Leite J.R., Alves P.B., Canton A.C., Teixeira-Silva F. (2010). Anxiolytic-like effect of sweet orange aroma in Wistar rats. Prog. Neuropsychopharmacol. Biol. Psychiatry.

[185] Dougnon G., Ito M. (2020). Sedative effects of the essential oil from the leaves of Lantana camara occurring in the Republic of Benin via inhalation in mice. J. Nat. Med..

[186] Takahashi M., Yoshino A., Yamanaka A., Asanuma C., Satou T., Hayashi S., Masuo Y., Sadamoto K., Koike K. (2012). Effects of Inhaled Lavender Essential Oil on Stress-Loaded Animals: Changes in Anxiety-Related Behavior and Expression Levels of Selected mRNAs and Proteins. Nat. Prod. Commun..

[187] Chioca L.R., Ferro M.M., Baretta I.P., Oliveira S.M., Silva C.R., Ferreira J., Losso E.M., Andreatini R. (2013). Anxiolytic-like effect of lavender essential oil inhalation in mice: Participation of serotonergic but not GABAA/benzodiazepine neurotransmission. J. Ethnopharmacol..

[188] Takahashi M., Yamanaka A., Asanuma C., Asano H., Satou T., Koike K. (2014). Anxiolytic-like Effect of Inhalation of Essential Oil from Lavandula officinalis: Investigation of Changes in 5-HT Turnover and Involvement of Olfactory Stimulation. Nat. Prod. Commun..

[189] Shaw D., Annett J.M., Doherty B., Leslie J.C. (2007). Anxiolytic effects of lavender oil inhalation on open-field behaviour in rats. Phytomed. Int. J. Phytother. Phytopharm..

[190] Bradley B.F., Starkey N.J., Brown S.L., Lea R.W. (2007). Anxiolytic effects of Lavandula angustifolia odour on the Mongolian gerbil elevated plus maze. J. Ethnopharmacol..

[191] Hawken P.A.R., Fiol C., Blache D. (2012). Genetic differences in temperament determine whether lavender oil alleviates or exacerbates anxiety in sheep. Physiol. Behav..

[192] Woelk H., Schläfke S. (2010). A multi-center, double-blind, randomised study of the Lavender oil preparation Silexan in comparison to Lorazepam for generalized anxiety disorder. Phytomedicine.

[193] Chen C.-J., Tseng Y.-H., Chu F.-H., Wen T.-Y., Cheng W.-W., Chen Y.-T., Tsao N.-W., Wang S.-Y. (2012). Neuropharmacological activities of fruit essential oil from Litsea cubeba Persoon. J. Wood Sci..

[194] Tankam J.M., Ito M. (2014). Sedative, anxiolytic and antidepressant-like effects of inhalation of the essential oil of *Ocimum gratissimum* L. from Cameroon in mice. Ournal Pharmacogn. Phytochem..

[195] Siengsukon C.F., Nelson E., Williams-Cooke C., Ludwig R., Beck E.S., Vidoni E.D., Mahnken J.D., Stevens S., Drerup M., Bruce J. (2020). Cognitive behavioral therapy for insomnia to enhance cognitive function and reduce the rate of Aβ deposition in older adults with symptoms of insomnia: A single-site randomized pilot clinical trial protocol. Contemp. Clin. Trials.

[196] Fultz N.E., Bonmassar G., Setsompop K., Stickgold R.A., Rosen B.R., Polimeni J.R., Lewis L.D. (2019). Coupled electrophysiological, hemodynamic, and cerebrospinal fluid oscillations in human sleep. Science.

[197] Xie L., Kang H., Xu Q., Chen M.J., Liao Y., Thiyagarajan M., O’Donnell J., Christensen D.J., Nicholson C., Iliff J.J. (2013). Sleep drives metabolite clearance from the adult brain. Science.

[198] Qiu H., Zhong R., Liu H., Zhang F., Li S., Le W. (2016). Chronic Sleep Deprivation Exacerbates Learning-Memory Disability and Alzheimer’s Disease-Like Pathologies in AβPP(swe)/PS1(ΔE9) Mice. J. Alzheimer’s Dis..

[199] Irwin M.R., Olmstead R., Carroll J.E. (2016). Sleep Disturbance, Sleep Duration, and Inflammation: A Systematic Review and Meta-Analysis of Cohort Studies and Experimental Sleep Deprivation. Biol. Psychiatry.

[200] Xu L., Li X., Zhang Y., Ding M., Sun B., Su G., Zhao Y. (2021). The effects of linalool acupoint application therapy on sleep regulation. RSC Adv..

[201] Yang H., Woo J., Pae A.N., Um M.Y., Cho N.-C., Park K.D., Yoon M., Kim J., Lee C.J., Cho S. (2016). α-Pinene, a Major Constituent of Pine Tree Oils, Enhances Non-Rapid Eye Movement Sleep in Mice through GABAA-benzodiazepine Receptors. Mol. Pharmacol..

[202] Woo J., Yang H., Yoon M., Gadhe C.G., Pae A.N., Cho S., Lee C.J. (2019). 3-Carene, a Phytoncide from Pine Tree Has a Sleep-enhancing Effect by Targeting the GABAA-benzodiazepine Receptors. Exp. Neurobiol..

[203] Sobreira Dantas Nóbrega de Figuêiredo F.R., Monteiro Á.B., Alencar de Menezes I.R., Sales V.D.S., Petícia do Nascimento E., Kelly de Souza Rodrigues C., Bitu Primo A.J., da Cruz L.P., do Nascimento Amaro É., de Araújo Delmondes G. (2019). Effects of the Hyptis Martiusii Benth. Leaf Essential Oil and 1,8-Cineole (Eucalyptol) on the Central Nervous System of Mice. Food Chem Toxicol.

[204] De Sousa D.P., de Farias Nóbrega F.F., de Almeida R.N. (2007). Influence of the chirality of (R)-(−)- and (S)-(+)-carvone in the central nervous system: A comparative study. Chirality.

[205] Herrmann F., Wink M. (2011). Synergistic interactions of saponins and monoterpenes in HeLa cells, Cos7 cells and in erythrocytes. Phytomedicine.

[206] Gertsch J. (2011). Botanical Drugs, Synergy, and Network Pharmacology: Forth and Back to Intelligent Mixtures. Planta Med..

[207] Eid S.Y., El-Readi M.Z., Eldin E.E.M.N., Fatani S.H., Wink M. (2013). Influence of combinations of digitonin with selected phenolics, terpenoids, and alkaloids on the expression and activity of P-glycoprotein in leukaemia and colon cancer cells. Phytomedicine.

[208] Jayamani J., Shanmugam G. (2014). Gallic acid, one of the components in many plant tissues, is a potential inhibitor for insulin amyloid fibril formation. Eur. J. Med. Chem..

[209] Yu M., Chen X., Liu J., Ma Q., Zhuo Z., Chen H., Zhou L., Yang S., Zheng L., Ning C. (2019). Gallic acid disruption of Aβ1-42 aggregation rescues cognitive decline of APP/PS1 double transgenic mouse. Neurobiol. Dis..

[210] Ehrnhoefer D.E., Bieschke J., Boeddrich A., Herbst M., Masino L., Lurz R., Engemann S., Pastore A., Wanker E.E. (2008). EGCG redirects amyloidogenic polypeptides into unstructured, off-pathway oligomers. Nat. Struct. Mol. Biol..

[211] Huang R., Vivekanandan S., Brender J.R., Abe Y., Naito A., Ramamoorthy A. (2012). NMR Characterization of Monomeric and Oligomeric Conformations of Human Calcitonin and Its Interaction with EGCG. J. Mol. Biol..

[212] Alternative Pathways of Human Islet Amyloid Polypeptide Aggregation Distinguished by 19F Nuclear Magnetic Resonance-Detected Kinetics of Monomer Consumption|Biochemistry. https://pubs.acs.org/doi/10.1021/bi3012548.

[213] Franko A., Rodriguez Camargo D.C., Böddrich A., Garg D., Rodriguez Camargo A., Rathkolb B., Janik D., Aichler M., Feuchtinger A., Neff F. (2018). Epigallocatechin gallate (EGCG) reduces the intensity of pancreatic amyloid fibrils in human islet amyloid polypeptide (hIAPP) transgenic mice. Sci. Rep..

[214] Ferreira N., Cardoso I., Domingues M.R., Vitorino R., Bastos M., Bai G., Saraiva M.J., Almeida M.R. (2009). Binding of epigallocatechin-3-gallate to transthyretin modulates its amyloidogenicity. FEBS Lett..

[215] Ferreira N., Pereira-Henriques A., Almeida M.R. (2015). Transthyretin chemical chaperoning by flavonoids: Structure-activity insights towards the design of potent amyloidosis inhibitors. Biochem. Biophys. Rep..

[216] Ferreira N., Saraiva M.J., Almeida M.R. (2011). Natural polyphenols inhibit different steps of the process of transthyretin (TTR) amyloid fibril formation. FEBS Lett..

[217] Garcia-Alloza M., Borrelli L.A., Rozkalne A., Hyman B.T., Bacskai B.J. (2007). Curcumin labels amyloid pathology in vivo, disrupts existing plaques, and partially restores distorted neurites in an Alzheimer mouse model. J. Neurochem..

[218] Ono K., Yamada M. (2006). Antioxidant compounds have potent anti-fibrillogenic and fibril-destabilizing effects for alpha-synuclein fibrils in vitro. J. Neurochem..

[219] Ghajarbeygi P., Hajhoseini A., Hosseini M.-S., Sharifan A. (2019). An In Vitro and In Vivo Cholinesterase Inhibitory Activity of *Pistacia khinjuk* and *Allium sativum* Essential Oils. J. Pharmacopuncture.

[220] Azimi M., Sharifan A., Tarzi B.G. (2017). The Use of Pistacia khinjuk Essential Oil to Modulate Shelf-Life and Organoleptic Traits of Mechanically Deboned Chicken Meat. J. Food Process. Preserv..

[221] Hawrył A., Hawrył M., Waksmundzka-Hajnos M. (2019). Liquid chromatography fingerprint analysis and antioxidant activity of selected lavender species with chemometric calculations. PLoS ONE.

[222] Amer Tawfeeq A., Faisal M., Abaas I., Alwan A. (2018). Isolation, quantification, and identification of rosmarinic acid, gas chromatography-mass spectrometry analysis of essential oil, cytotoxic effect, and antimicrobial investigation of rosmarinus officinalis leaves. Asian J. Pharm. Clin. Res..

[223] Boiangiu R.S., Brinza I., Hancianu M., Erdogan Orhan I., Eren G., Gündüz E., Ertas H., Hritcu L., Cioanca O. (2020). Cognitive Facilitation and Antioxidant Effects of an Essential Oil Mix on Scopolamine-Induced Amnesia in Rats: Molecular Modeling of In Vitro and In Vivo Approaches. Molecules.

[224] Armijos C., Matailo A., Bec N., Salinas M., Aguilar G., Solano N., Calva J., Ludeña C., Larroque C., Vidari G. (2020). Chemical composition and selective BuChE inhibitory activity of the essential oils from aromatic plants used to prepare the traditional Ecuadorian beverage horchata lojana. J. Ethnopharmacol..

[225] Bailon-Moscoso N., Tinitana F., Martínez-Espinosa R., Jaramillo-Velez A., Palacio-Arpi A., Aguilar-Hernandez J., Romero-Benavides J.C. (2017). Cytotoxic, antioxidative, genotoxic and antigenotoxic effects of Horchata, beverage of South Ecuador. BMC Complement. Altern. Med..

[226] Tene V., Malagon O., Vita-Finzi P., Vidari G., Armijos C., Zaragoza T. (2007). An ethnobotanical survey of medicinal plants used in Loja and Zamora-Chinchipe, Ecuador. J. Ethnopharmacol..

[227] Wagner H. (2006). Multitarget therapy--the future of treatment for more than just functional dyspepsia. Phytomed. Int. J. Phytother. Phytopharm..

[228] Kennedy D.O., Scholey A.B., Wesnes K.A. (2001). Differential, dose dependent changes in cognitive performance following acute administration of a Ginkgo biloba/Panax ginseng combination to healthy young volunteers. Nutr. Neurosci..

[229] Butterweck V., Petereit F., Winterhoff H., Nahrstedt A. (1998). Solubilized hypericin and pseudohypericin from Hypericum perforatum exert antidepressant activity in the forced swimming test. Planta Med..

[230] Wagner H., Ulrich-Merzenich G. (2009). Synergy research: Approaching a new generation of phytopharmaceuticals. Phytomed. Int. J. Phytother. Phytopharm..

[231] Rezai-Zadeh K., Shytle D., Sun N., Mori T., Hou H., Jeanniton D., Ehrhart J., Townsend K., Zeng J., Morgan D. (2005). Green tea epigallocatechin-3-gallate (EGCG) modulates amyloid precursor protein cleavage and reduces cerebral amyloidosis in Alzheimer transgenic mice. J. Neurosci. Off. J. Soc. Neurosci..

[232] Ferreira N., Saraiva M.J., Almeida M.R. (2012). Epigallocatechin-3-gallate as a potential therapeutic drug for TTR-related amyloidosis: “in vivo” evidence from FAP mice models. PLoS ONE.

[233] Bagci E., Aydin E., Ungureanu E., Hritcu L. (2016). *Anthriscus nemorosa* essential oil inhalation prevents memory impairment, anxiety and depression in scopolamine-treated rats. Biomed. Pharmacother..

[234] Maleki H., Norouzi R., Shahi A. (2020). Essential oil components, phenolic content and antioxidant activity of Anthriscus cerefolium and Anthriscus sylvestris from Iran. J. Hortic. Postharvest Res..

[235] Areias F., Valentão P., Andrade P., Moreira M., Amaral J., Seabra R. (2000). HPLC/DAD analysis of phenolic compounds from lavender and its application to quality control. J. Liq. Chromatogr. Relat. Technol..

[236] López V., Nielsen B., Solas M., Ramírez M.J., Jäger A.K. (2017). Exploring Pharmacological Mechanisms of Lavender (*Lavandula angustifolia*) Essential Oil on Central Nervous System Targets. Front. Pharmacol..

[237] Ko L.-W., Su C.-H., Yang M.-H., Liu S.-Y., Su T.-P. (2021). A pilot study on essential oil aroma stimulation for enhancing slow-wave EEG in sleeping brain. Sci. Rep..

[238] Velasco-Rodríguez R., Pérez-Hernández M.G., Maturano-Melgoza J.A., Hilerio-López Á.G., Monroy-Rojas A., Arana-Gómez B., Vásquez C. (2019). The effect of aromatherapy with lavender (*LLavandula angustifolia*L) on serum melatonin levels. Complement. Ther. Med..

[239] Sadiki F.Z., Idrissi M.E., Cioanca O., Trifan A., Hancianu M., Hritcu L., Postu P.A. (2019). Tetraclinis articulata essential oil mitigates cognitive deficits and brain oxidative stress in an Alzheimer’s disease amyloidosis model. Phytomedicine.

[240] Harouak H., Ibijbijen J., Nassiri L. (2021). Chemical profile of *Tetraclinis articulata* (Vahl) Masters, and *Juglans regia* L. and *Olea europaea* L. var. *Sylvestris* used against oral diseases: In Vitro analysis between polyphenolic content and aqueous extraction optimization. Heliyon.

